# Exploring the efficacy of PARP inhibitors in metastatic castration-resistant prostate cancer with homologous recombination repair alteration: a meta-analysis based on subgroups and reconstructed individual patient data

**DOI:** 10.1097/JS9.0000000000003338

**Published:** 2025-09-19

**Authors:** Fuxun Zhang, Zhirong Luo, Yang Xiong, Qi Xue, Xuyan Guo, Qiang Fu, Yong Jiao, Wei Zhang, Pati-Alam Alisha, Uzoamaka Adaobi Okoli, Geng Zhang

**Affiliations:** a Department of Urology, Tangdu Hospital, Fourth Military Medical University, Xi’an, Shaanxi, China; b Division of Surgery & Interventional Science, University College London, London, UK; c Department of Urology, Institute of Urology, West China Hospital, Sichuan University, Chengdu, Sichuan, China; d Basic and Translational Cancer Research Group, Department of Pharmacology and Therapeutics, College of Medicine, University of Nigeria, Nsukka, Enugu State, Nigeria

**Keywords:** individual patient data, poly (ADP-ribose) polymerase inhibitor, prostate cancer, survival, treatment

## Abstract

**Background:**

Treatment for metastatic castration-resistant prostate cancer (mCRPC) harboring homologous recombination repair (HRR) alteration remains a challenge. Recently published trials have evaluated the poly (ADP-ribose) polymerase inhibitors (PARPIs) in mCRPC. However, the efficacy in subgroup with specific HRR gene mutation and treatment protocol requires further elucidation. This meta-analysis aims to explore the efficacy of PARPIs based on subgroups and reconstructed individual patient data (IPD).

**Methods:**

Literature was searched using PubMed, Embase, Cochrane Library, and ClinicalTrials.gov up to April 2025. The primary outcome was radiographic progression-free survival (rPFS), and the secondary outcomes included overall survival (OS), prostate-specific antigen progression-free survival (PSA-PFS), and adverse events (AEs). Hazard ratios (HRs) and risk ratios (RRs) were pooled as the indicators using inverse-variance and Mantel-Haenszel methods. IPD was reconstructed from Kaplan–Meier curve. Survival analysis was performed using Cox proportional hazards model based on the reconstructed IPD. Heterogeneity was assessed by *I^2^* and sensitivity analysis. Publication bias was examined via contour‑enhanced funnel plots.

**Results:**

Data of 1840 mCRPC patients with HRR alteration from five pivotal phase III clinical trials were analyzed. PARPIs significantly improved overall rPFS (HR: 0.55) and OS (HR: 0.85). PARPIs also prolonged rPFS across the subgroups defined by clinicopathologic features. In*BRCA1/2* subgroup, survival benefits were prominent for rPFS (HR 0.32) and OS (HR 0.70). For patients with non-*BRCA* alterations, no benefits of PARPIs were detected for rPFS and OS in *ATM* subgroup, and for OS in *CDK12* subgroup. Survival analyses indicated that PARPIs treatment was significantly associated with the improved rPFS (HR: 0.73, *P* < 0.001) and PSA-PFS (HR: 0.80, *P* = 0.020) in the overall population, and revealed OS benefit in *BRCA1/2* subgroup (HR: 0.77, *P* = 0.030). Comparing with monotherapy, combination regimen of PARPIs provided greater benefits for rPFS (HR: 0.56, *P* < 0.001)and OS (HR: 0.64., *P* < 0.001).

**Conclusions:**

PARPIsimprove survival in mCRPC patients with *BRCA1/2* mutation, but have no effect in those with *ATM* mutation. Comparing with PARPIs monotherapy, the combination regimen provides greater survival benefit in the overall population. Future investigation should validate these findings in real-world settings.

## Introduction

Prostate cancer (PCa) is the second most common malignancy in men^[1]^. It is estimated that 1 466 680 PCa cases were diagnosed, and 396 792 died in 2022 worldwide^[1,2]^. Over 100 000 men in the United States were reportedly living with metastatic PCa, and this number is expected to reach approximately 192 500 by 2023^[3]^. Currently, a multidisciplinary team of urologists and oncologists could provide various treatment regimens for metastatic PCa patients, including androgen deprivation therapy (ADT) and chemotherapy^[4–6]^. The 5-year survival of patients with metastatic PCa is 32% with the development of treatment strategies^[7]^. However, the progression to metastatic castration-resistant prostate cancer (mCRPC), an incurable end stage of PCa, is almost inevitable in most cases^[8]^.

In recent years, multiple pivotal clinical trials have led to novel drug approvals, making several emerging therapies available for mCRPC patients, such as poly(ADP-ribose) polymerase inhibitors (PARPIs), novel androgen receptor pathway inhibitors (ARPIs) and radioligand therapy^[6,9,10]^. Among them, PARPIs targeting to inhibit deoxyribonucleic acid (DNA) damage-repairing pathway are lethal for PCa harboring homologous recombination repair (HRR) gene mutations^[11]^. It is reported that approximately 30.6% mCRPC patients have HRR mutation, including 13.2% *Breast Cancer 1/2* (*BRCA1/2*), and 17.4% non-*BRCA* mutation, such as *Ataxia Telangiectasia Mutated* (*ATM), Cyclin-Dependent Kinase 12* (*CDK12*), and *Checkpoint Kinase 2* (*CHEK2*)^[12,13]^. It is important to emphasize that patients with HRR alteration may have worse clinical outcomes^[13,14]^. Thus, personalized treatment options for specific subgroups are crucial to improve the prognosis of mCRPC.

The efficacy and safety of several PARPIs have been evaluated in recently published phase III clinical trials, including PROfound, PROpel, MAGNITUDE, TALAPRO-2, and TRITON3^[4,5,15–21]^. These trials demonstrated that PARPIs treatment significantly improved the prognosis of mCRPC patients with HRR alteration. However, subgroups of specific clinical characteristics and HRR gene mutations had relatively small sample sizes and event counts, and the treatment regimens of PARPIs were different. Therefore, we comprehensively explore the efficacy and safety of PARP based on subgroups with specific HRR gene mutation and clinical characteristics and reconstructed individual-level data from pivotal phase III randomized controlled trials (RCTs). This meta-analysis is compliant to the TITAN Guidelines 2025 for the transparency in AI use^[22]^.

## Methods

### Literature search

This meta-analysis was conducted according to the PRISMA, and has been reported in line with AMSTAR guidelines^[23,24]^. Studies were searched using PubMed, Embase, and Cochrane Library from inception to April 2025. Search strategy included: “Prostate neoplasms” OR “Prostate cancer” OR “metastatic prostate cancer” OR “Castration-resistant prostate cancer” AND “Poly (ADP-ribose) polymerases” OR “Poly (ADP-ribose) synthase” OR “PARP” OR “Poly (ADP-ribose) polymerase inhibitor” OR “PARP inhibitor.” Additional clinical trials were searched manually in the ClinicalTrials.gov according to search terms, and any disagreements were resolved with a third reviewer. The specific protocol of this study was submitted to PROSPERO (CRD 420251042411).

### Literature selection

The inclusion criteria of this meta-analysis are as follows: (a) Prostate adenocarcinoma was diagnosed histologically. (b) Diagnosis of mCRPC was established. (c) Published phase III RCTs, including interim and final prespecified analysis. (d) Studies containing HRR alteration population and PARPIs cohort. (e) Studies reporting survival outcomes with Kaplan–Meier curves. (f) Studies published in English. The background therapy, including ARPIs or docetaxel, was not restricted. Studies without reporting survival curve were excluded. Retrospective studies, reviews, commentaries, case reports, and letters were excluded.

### Data extraction

This study was conducted based on HRR alteration population and intention-to-treat (ITT) basis. The most recent data from published phase III RCTs of PARPIs were extracted for meta-analysis. Extracted data included baseline characteristics, treatment protocols, ITT sizes, event counts, event ratios, and survival curves. For each study, updated event counts and ratios were extracted from the most recent publication unadjusted by crossover design, and the data from crossover cohort was excluded. All data were extracted from the most current reports of clinical trials. If survival data were immature, the results from most recent interim analysis were used with appropriate caution. If the subgroup data were not reported in the final prespecified analysis, they were extracted from previous publication. When data were analyzed by both blinded independent central review and investigator, the data analyzed by central review were selected as priority. Two authors (ZL and YX) independently reviewed and extracted the data, while two additional authors (QX and XG) were designated to identify these data, and any discrepancies in values or time points were resolved by consensus.

### IPD reconstruction

Given the original researches provided Kaplan–Meier curves rather than individual patient data (IPD), we reconstructed IPD using the inverted Kaplan–Meier method as previously reported^[25,26]^. We obtained the data from survival curves using Engauge Digitizer (version 12.1). Each Kaplan–Meier plot was imported into Engauge Digitizer to extract the coordinates of time and survival probability at finely spaced intervals, and to capture all reported numbers at risk. The IPD of time-to-event outcomes were simulated based on coordinates, event counts, and numbers at risk by using IPDfromKM package. This method allowed us to obtain patient-level survival data from published survival curves when original IPD were uneasily available, reconstructing accurate survival distribution for further analyses.

### Outcomes and quality assessment

The primary outcome of this meta-analysis was radiographic progression-free survival (rPFS). The secondary outcomes included overall survival (OS), prostate-specific antigen progression-free survival (PSA-PFS) and the adverse events (AEs) without specifying symptoms. In this regard, survival outcomes were defined as the interval from randomization to event or death from any cause. The quality assessment of studies was performed by two independent reviewers (QF and WZ) based on Cochrane Risk of Bias 2 (RoB2) tool^[27]^. Five different domains of RoB2 tool include randomization process, deviations from intended intervention, missing outcome data, measurement of the outcome, and selection of the reported result. The RoB for each domain and overall assessment was categorized as low, some concerns, or high RoB. Any disagreement in this section was discussed until a consensus was achieved.

### Statistical analysis

All analyses were performed on the basis of the full analysis set and ITT population with HRR alteration. The natural logarithm of the hazard ratio (HR) was used as the effect size (ES) for both OS and PFS. If specific HR were not reported directly, it was estimated from the published results, including event counts and sample size, or synthesized using an inverse-variance weighted fixed-effect model^[28]^.

Pooled estimates of logHR were obtained using the inverse-variance method under both fixed effect and random-effects models. Variance (τ^2^) between studies in the random effects model was estimated via the restricted maximum likelihood (REML) estimator. Safety was evaluated using risk ratios (RRs) with 95% confidence intervals (CIs), calculated from the number of events and total patients per arm. RRs were pooled using the Mantel-Haenszel method under the fixed-effect model, and using the inverse variance method with τ^2^ estimated by the REML estimator under the random effects model. Continuity correction of 0.5 was applied for studies with zero event cells. Cochran’s Q statistic was used to assess the differences between subgroups. Overall heterogeneity was evaluated by the Cochran’s Q test and quantified with *I^2^* test. Confidence intervals for τ^2^ and τ were calculated using the Q Profile method. *I^2^* of < 30%, 30% ≤ *I^2^* < 50%, 50% ≤ *I^2^* < 75%, and *I^2^* ≥ 75% indicated low, moderate, substantial, and considerable heterogeneity, respectively. In this meta-analysis, only the random effects results were reported.

To evaluate the influence of individual studies on the overall estimates, a leave-one-out sensitivity analysis was performed when synthesized studies were three or more, with graphical representation of each omitted-study effect alongside the pooled logHR. Meta-regression was also applied to explore the impact of variables on outcomes. Publication bias was examined by visual inspection of contour-enhanced funnel plots when included studies were three or more. The null effect contour is defined as the contour drawn at a specified confidence level (90%, 95%, and 99%) centered on the null hypothesis of no effect (log(HR) = 0).

Survival analysis was performed using Cox proportional hazards regression and Kaplan–Meier survival estimation with log-rank test. The association between groups and survival was assessed using the Cox model with hazard ratio (HR) and 95%CIs, and the log-rank test was applied to compare. Meanwhile, primary potential confounding factors, including background therapy and trials, were adjusted for in Cox proportional hazards regression model. The Review Manager software and R 4.4.2 (survival, survminer, IPDfromKM, and ggplot2 packages) were used.

## Results

### Characteristics of the included studies

**Figure 1.**
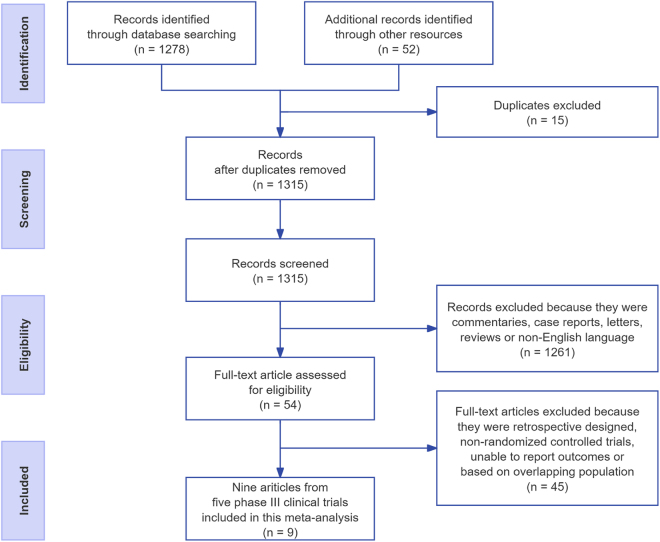
Flowchart of searching literature.

The flowchart of literature selection is shown in Figure 1. A total of 1315 studies were retrieved after duplicates were removed, and 54 studies were assessed for inclusion eligibility. Finally, nine published studies from five phase III clinical trials, PROfound (Olaparib, NCT02987543), PROpel (Olaparib, NCT03732820), MAGNITUDE (Niraparib, NCT03748641), TALAPRO-2 (Talazoparib, NCT03395197) and TRITON3 (Rucaparib, NCT02975934), were included^[4,5,15–21]^. The data of 1840 patients in HRR alteration population were extracted and analyzed eventually. The detailed characteristics of included studies are shown in Table 1. The RoB summary of included studies is demonstrated in Supplemental Digital Content Figure S1, available at: http://links.lww.com/JS9/F126.

**Table 1:** Baseline characteristics of included studies

Trial	Register No.	Authors	Publication date	Publication journal	Title	Stage	Design	Baseline median age (year)	ITT with PARPIs	ITT with control
PROfound^[15,16]^	NCT02987543	de Bono J, et al.	2020	N Engl J Med	Olaparib for metastatic castration-resistant prostate cancer	Final analysis	Olaparib *vs* Physician’s choice (enzalutamide or abiraterone)	69	256	131
		Hussain M et al.	2020	N Engl J Med	Survival with olaparib in metastatic castration-resistant prostate cancer					
PROpel^[4,17]^	NCT03732820	Clarke NW, et al.	2022	NEJM Evid	Abiraterone and olaparib for metastatic castration-resistant prostate cancer	Finalanalysis	Olaparib *vs* Placebo, with background therapy of abiraterone	69	111	115
		Saad F, et al.	2023	Lancet Oncol	Olaparib plus abiraterone versus placebo plus abiraterone in metastatic castration-resistant prostate cancer (PROpel): final prespecified overall survival results of a randomized, double-blind, phase 3 trial					
MAGNITUDE^[5,18]^	NCT03748641	Chi KN, et al.	2023	J Clin Oncol	Niraparib and abiraterone acetate for metastatic castration-resistant prostate cancer	OS is immature	Niraparib *vs* placebo, with background therapy of abiraterone	69	212	211
		Chi KN, et al.	2023	Ann Oncol	Niraparib plus abiraterone acetate with prednisone in patients with metastatic castration-resistant prostate cancer and homologous recombination repair gene alterations: second interim analysis of the randomized phase III MAGNITUDE trial					
TRITON3^[21]^	NCT02975934	Fizazi K, et al.	2023	N Engl J Med	Rucaparib or physician’s choice in metastatic prostate cancer	OS is immature	Rucaparib *vs* physician’s choice (docetaxel or abiraterone acetate or enzalutamide)	71	270	135
TALAPRO-2^[19,20]^	NCT03395197	Agarwal N, et al.	2023	Lancet	Talazoparib plus enzalutamide in men with first-line metastatic castration-resistant prostate cancer (TALAPRO-2): a randomized, placebo-controlled, phase 3 trial	OS is immature	Talazoparib* vs* placebo, with background therapy of enzalutamide	71	200	199
		Fizazi K, et al.	2024	Nat Med	First-line talazoparib with enzalutamide in HRR-deficient metastatic castration-resistant prostate cancer: the phase 3 TALAPRO-2 trial					

ITT: Intent-to-treat; NCT: National clinical trial; OS: Overall survival; PARPIs: Poly(ADP-ribose) polymerase inhibitors.

ITT refer to population with homologous recombination repair alteration.

### The efficacy of PARPIs in the overall population with HRR alteration

**Figure 2.**
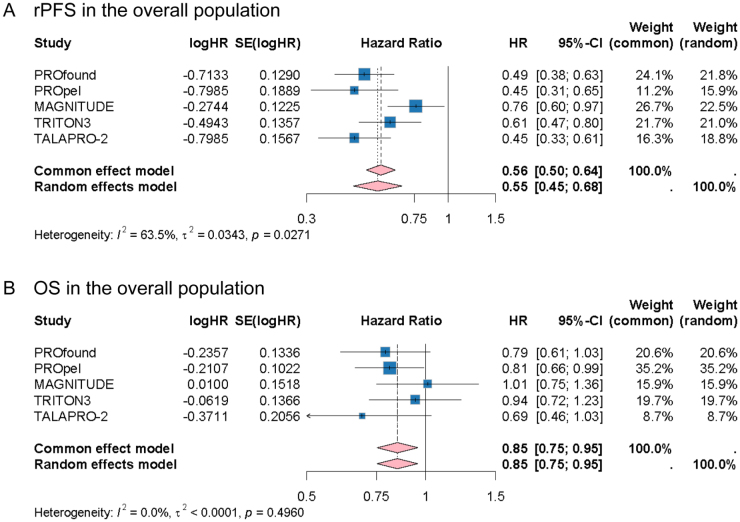
Forest plots showing the effects of PARPIs on rPFS (A) and OS (B) in the overall population with HRR alteration.

Abbreviations: CI: Confidence intervals; HR: Hazard ratio; HRR: Homologous recombination repair; OS: Overall survival; PARPIs: Poly(ADP-ribose) polymerase inhibitors; rPFS: Radiographic progression-free survival.

The log-HRs for PFS and OS were pooled to assess the efficacy of PARPIs in the mCRPC population with HRR alterations. There was substantial heterogeneity in pooled rPFS (HR: 0.55, 95% CI: 0.45–0.68, *I^2^* = 63.5%, *P* = 0.0271) (Fig. 2A). Sensitivity analyses identified the MAGNITUDE trial as the principal source of heterogeneity (Supplemental Digital Content Figure S2A, available at: http://links.lww.com/JS9/F126). The heterogeneity was eliminated when excluding it (HR: 0.51, 95% CI: 0.44–0.59, *I^2^* = 0.0%, *P* = 0.4064) or using the rPFS by investigator review (HR: 0.53, 95% CI: 0.46–0.60, *I^2^* = 9.3%, *P* = 0.3534) (Supplemental Digital Content Figure S3A,B, available at: http://links.lww.com/JS9/F126). Sensitivity analyses revealed consistent low heterogeneity when MAGNITUDE was excluded or investigator-reviewed (Supplemental Digital Content Figure S2B,C, available at: http://links.lww.com/JS9/F126). Compared with control, PARPIs treatment was associated with improved the rPFS in mCRPC (HR: 0.51–0.55).

Moreover, PARPIs treatment significantly prolonged the OS in HRR alteration overall population with mCRPC (HR: 0.85, 95% CI: 0.75–0.95, *I^2^* = 0.0%, *P* = 0.4960) (Fig. 2B). In leave-one-out sensitivity analyses, omitting any single study produced minimal change in the pooled log-HR (Supplemental Digital Content Figure S2D, available at: http://links.lww.com/JS9/F126). Additionally, despite the considerable heterogeneity, consistent direction and interval of pooled PSA-PFS revealed the effect of PARP on improving PSA-PFS (HR: 0.51, 95% CI: 0.34–0.78, *I^2^* = 76.6%, *P* = 0.0389) (Supplemental Digital Content Figure S3C, available at: http://links.lww.com/JS9/F126). In meta-regression, the patient age, PARPIs type (Olaparib *vs* Non-olaparib) and background therapy (PARPIs monotherapy *vs* PARPIs plus ARPIs) did not show a statistically significant influence on rPFS and OS (All *P* > 0.05) (Supplemental Digital Content Table S1, available at: http://links.lww.com/JS9/F127). Among them, PARPIs type accounted for about 17.6% of the heterogeneity (R^2^: 17.63%), and other PARPIs trended to higher HR comparing with olaparib in rPFS (β = 0.244). However, this trend was not significant (*P* > 0.05) (Supplemental Digital Content Table S1, available at: http://links.lww.com/JS9/F127).

### The efficacy of PARPIs in subgroups defined by clinical characteristics

**Figure 3.**
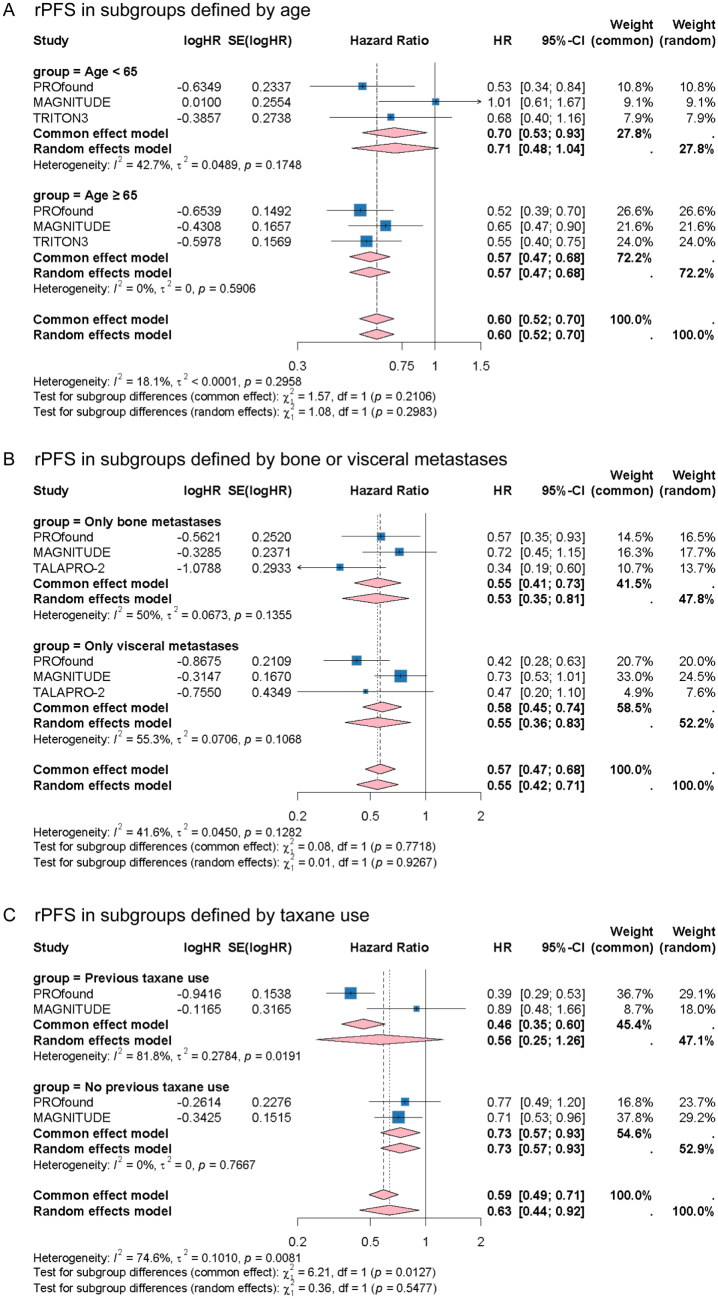
Forest plots showing the effects of PARPIs on rPFS in subgroups defined by age (A), bone or visceral metastases (B) and previous taxane use(C).

Abbreviations: CI: Confidence intervals; HR: Hazard ratio; PARPIs: Poly(ADP-ribose) polymerase inhibitors; rPFS: Radiographic progression-free survival.

PARPIs treatment prolonged the rPFS across subgroups at age < 65 and age ≥ 65 (HR: 0.71, 95% CI: 0.48–1.04, *I^2^* = 42.7%, *P* = 0.1748 and HR: 0.57, 95% CI: 0.47–0.68, *I^2^* = 0.0%, *P* = 0.5906, respectively) (Fig. 3A). Although the heterogeneity was moderate, no significant difference was observed between subgroups at age < 65 and age ≥ 65 (*P* for subgroup differences = 0.2983). Moreover, leave-one-out sensitivity analyses indicated that the heterogeneity may attribute to the MAGNITUDE trial, and showed the robustness of the findings in age ≥ 65 subgroup (Supplemental Digital Content Figure S4A,B, available at: http://links.lww.com/JS9/F126). Meanwhile, PARPIs improved the rPFS in subgroups with bone metastasis and visceral metastasis comparing with controls (HR: 0.53, 95% CI: 0.35–0.81, *I^2^* = 50.0%, *P* = 0.1355 and HR: 0.55, 95% CI: 0.36–0.83, *I^2^* = 55.3%, *P* = 0.1068, respectively) (Fig. 3B). The difference between subgroups is insignificant (*P* for subgroup differences = 0.9267). Moreover, the direction of trials estimates were consistent in sensitivity analyses (Supplemental Digital Content Figure S4C,D, available at: http://links.lww.com/JS9/F126).

For patients with prior taxane use, the risk of radiographic progression in patients with PARPIs was reduced by 44% comparing with control group (HR: 0.56, 95% CI: 0.25–1.26, *I^2^* = 81.8%, *P* = 0.0191)(**Fig. 3C**). However, considerable heterogeneity was detected (*I^2^* = 81.8%). For patients with no taxane use, PARPIs treatment improved rPFS (HR: 0.73, 95% CI: 0.57–0.93, *I^2^* = 0.0%, *P* = 0.7667). No statistically significant difference was found between the pooled estimates for two subgroups (*P* for subgroup differences = 0.5477). Moreover, PARPIs prolonged the rPFS in patients with Eastern Cooperative Oncology Group (ECOG) score 0 (HR: 0.60, 95% CI: 0.50–0.72, *I^2^* = 0.0%, *P* = 0.6878) (Supplemental Digital Content Figure S5A, available at: http://links.lww.com/JS9/F126). The pooled estimate of rPFS in patients with ECOG score 1 was 0.54 with high heterogeneity (HR: 0.54, 95% CI: 0.40–0.74, *I^2^* = 55.1%, *P* = 0.0825) (Supplemental Digital Content Figure S5A, available at: http://links.lww.com/JS9/F126). No significant difference was observed between the pooled estimates for the ECOG score 0 or 1 subgroups (*P* for subgroup differences = 0.5840). The sensitivity analyses demonstrated consistency in ECOG score 0 subgroup, and low heterogeneity when omitting the MAGNITUDE in ECOG score 1 subgroup (Supplemental Digital Content Figure S5B,C, available at: http://links.lww.com/JS9/F126). After heterogeneous trials removed, the pooled rPFS for patients with ECOG score 1 was 0.48 (HR: 0.48, 95% CI: 0.38–0.60, *I^2^* = 0.0%, *P* = 0.5018) (Supplemental Digital Content Figure S6A, available at: http://links.lww.com/JS9/F126).

Additionally, the efficacy of PARPIs for rPFS in patients from different regions was also explored. The risk of radiographic progression in patients with PARPIs from Asia, Europe, and America were 66% (HR: 0.66, 95% CI: 0.46–0.94, *I^2^* = 0.0%, *P* = 0.9036), 63% (HR: 0.63, 95% CI: 0.37–1.07, *I^2^* = 76.3%, *P* = 0.0399), and 49% (HR: 0.49, 95% CI: 0.32–0.73, *I^2^* = 0.0%, *P* = 0.4464) comparing with controls (Supplemental Digital Content Figure S6B, available at: http://links.lww.com/JS9/F126). No subgroup differences were detected in analyses (*P* for subgroup differences = 0.5160).

### The efficacy of PARPIs on PFS in subgroups defined by mutated HRR gene

**Figure 4.**
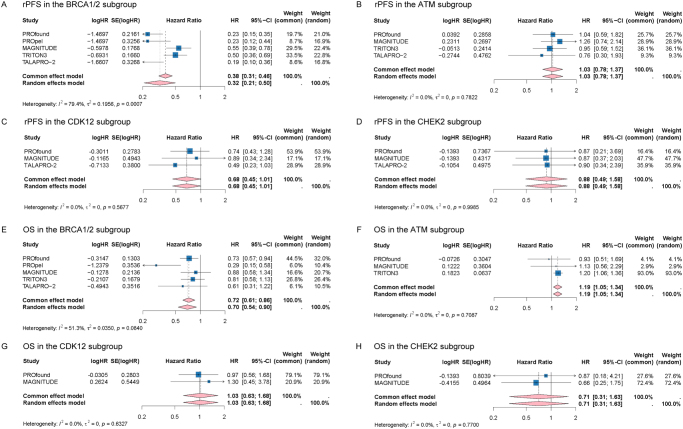
Forest plots showing the effects of PARPIs on rPFS in subgroups with *BRCA1/2* mutation (A), *ATM* mutation (B), *CDK12* mutation (C) and *CHEK2* mutation (D); The effects of PARPIs on OS in subgroups with *BRCA1/2* mutation (E), *ATM* mutation (F), *CDK12* mutation (G) and *CHEK2* mutation (H).

Abbreviations: ATM: Ataxia Telangiectasia Mutated gene; BRCA1/2: Breast Cancer 1/2 gene; CDK12: Cyclin-Dependent Kinase 12 gene; CHEK2: Checkpoint Kinase 2 gene; CI: Confidence intervals; OS: Overall survival; PARPIs: Poly(ADP-ribose) polymerase inhibitors; rPFS: Radiographic progression-free survival.

In patients with *BRCA1/2* mutation, PARPIs treatment was significantly associated with improved rPFS comparing with controls (HR: 0.32, 95% CI: 0.21–0.50, *I^2^* = 79.4%, *P* = 0.0007) (Fig. 4A). Due to considerable heterogeneity was observed, the subgroup analyses of PARPIs type (olaparib *vs* non-olaparib) in *BRCA1/2* patients were conducted. Significant efficacy of PARPIs with low heterogeneity was observed in *BRCA1/2* patients with olaparib (HR: 0.23, 95% CI: 0.16–0.33, *I^2^* = 0.0%, *P* = 1.0000), and no difference was detected between *BRCA1/2* patients with olaparib and those with non-olaparib PARPIs (*P* for subgroup differences = 0.1300) (Supplemental Digital Content Figure S7A, available at: http://links.lww.com/JS9/F126). Meanwhile, the consistent direction and interval of estimates in the sensitivity analyses support the efficacy of PARPIs in *BRCA1/2* patients (Supplemental Digital Content Figure S8A, available at: http://links.lww.com/JS9/F126).

For patients with non-*BRCA* HRR genes alteration, including *ATM, CDK12* and *CHEK2*, the pooled estimates for rPFS were 1.03 (HR: 1.03, 95% CI: 0.78-1.37, *I^2^* = 0.0%, *P* = 0.7822), 0.68 (HR: 0.68, 95% CI: 0.45–1.01, *I^2^* = 0.0%, *P* = 0.5677) and 0.88 (HR: 0.88, 95% CI: 0.49–1.58, *I^2^* = 0.0%, *P* = 0.9985), respectively (Fig. 4B-D). Sensitivity analyses showed that excluding any single estimate from the *ATM, CDK12* or *CHEK2* subgroups did not alter the overall outcomes (Supplemental Digital Content Figure S8B-D, available at: http://links.lww.com/JS9/F126). Additionally, the efficacy of PARPIs on PSA-PFS in in *BRCA1/2* subgroup was also explored (HR: 0.34, 95% CI: 0.15–0.75, *I^2^* = 88.0%, *P* = 0.0002) (Supplemental Digital Content Figure S7B, available at: http://links.lww.com/JS9/F126). In leave-one-out sensitivity analyses, the considerable heterogeneity was attributed to the PROpel which has small sample size of *BRCA1/2* subgroup (Supplemental Digital Content Figure S8E, available at: http://links.lww.com/JS9/F126). The risk of PSA progression was reduced by 50% (HR: 0.50, 95% CI: 0.40–0.64, *I^2^* = 0.0%, *P* = 0.7461) comparing with control group when excluding the PROpel (Supplemental Digital Content Figure S7C, available at: http://links.lww.com/JS9/F126).

### The efficacy of PARPIs on OS in subgroups defined by mutated HRR gene

In patients with *BRCA1/2* mutation, primarily pooled estimate for OS was 0.70 (HR: 0.70, 95% CI: 0.54–0.90, *I^2^* = 51.3%, *P* = 0.0840) (Fig. 4E). The sensitivity analyses demonstrated that substantial heterogeneity was attributed to the PROpel trial (Supplemental Digital Content Figure S8F, available at: http://links.lww.com/JS9/F126). The pooled estimate of OS was 0.77 and the heterogeneity was abolished after excluding the PROpel (HR: 0.77, 95% CI: 0.64–0.92, *I*^2^ = 0.0%*, P*= 0.7801) (Supplemental Digital Content Figure S7D, available at: http://links.lww.com/JS9/F126) Fig. 4E. The pooled estimates for OS in the *ATM, CDK12* and *CHEK2* subgroups were 1.19 (HR: 1.19, 95% CI: 1.05–1.34, *I^2^* = 0.0%, *P* = 0.7087), 1.03 (HR: 1.03, 95% CI: 0.63–1.68, *I^2^* = 0.0%, *P* = 0.6327) and 0.71 (HR: 0.71, 95% CI: 0.31–1.63, *I^2^* = 0.0%, *P* = 0.7700), respectively (Fig. 4F-H). The directions of estimates in the *ATM* subgroup were consistent (Supplemental Digital Content Figure S8G, available at: http://links.lww.com/JS9/F126).

### Survival analysis of reconstructed IPD data

**Figure 5.**
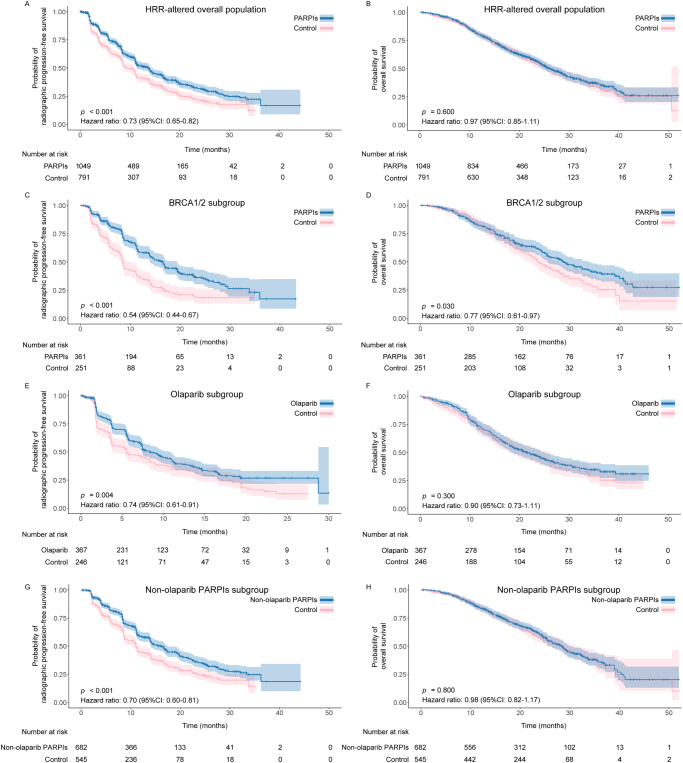
Kaplan-Meier curves of rPFS (A) and OS (B) in HRR-altered overall population treated with PARPIs versus control; Kaplan-Meier curves of rPFS (C) and OS (D) in *BRCA1/2* subgroup treated with PARPIs versus control; Kaplan-Meier curves of rPFS (E) and OS (F) in subgroup treated with olaparib versus control; Kaplan-Meier curves of rPFS (G) and OS (H) in subgroup treated with non-olaparib PARPIs versus control.

Abbreviations: BRCA1/2: Breast Cancer 1/2 gene; CI: Confidence intervals; HR: Hazard ratio; HRR: Homologous recombination repair; OS: Overall survival; PARPIs: Poly(ADP-ribose) polymerase inhibitors; rPFS: Radiographic progression-free survival.

Survival analyses for PFS and OS were performed using reconstructed IPD data. The Kaplan–Meier curves showed significant differences of rPFS and PSA-PFS between PARPIs and control groups in the overall population with HRR alteration, whereas no significant OS difference was observed. Among them, PARPIs treatment was significantly associated with improved rPFS (HR: 0.73, 95% CI: 0.65–0.82, *P* < 0.001) (Fig. 5A), and PSA-PFS (HR: 0.80, 95% CI: 0.66–0.97, *P* = 0.020) (Supplemental Digital Content Figure S9A, available at: http://links.lww.com/JS9/F126). The OS benefit of PARPIs was not detected in the overall population (HR: 0.97, 95% CI: 0.85–1.11, *P* = 0.600) (Fig. 5B).

Please note: Reconstructed IPD for HRR-altered overall population, olaparib subgroup and non-PARPIs subgroup were extracted from all five included trials; Reconstructed IPD for *BRCA1/2* subgroup were extracted from PROpel, MAGNITUDE, and TRITON3; OS data of MAGNITUDE, TALAPRO-2, and TRITON3 are immature.

PFS and OS benefits in subgroups with *BRCA1/2* mutation or treated by olaparib and non-olaparib PARPIs were evaluated. For *BRCA1/2* subgroup, PARPIs treatment significantly improved the rPFS (HR: 0.54, 95% CI: 0.44–0.67, *P* < 0.001) (Fig. 5C). Meanwhile, long-term OS benefit of PARPIs was also observed in the *BRCA1/2* subgroup (Fig. 5D). On the other hand, olaparib improved the rPFS comparing with controls (HR: 0.74, 95% CI: 0.61–0.91, *P* = 0.004), but the effect of olaparib on OS tended to be null (HR: 0.90, 95% CI: 0.73–1.11, *P* = 0.300) (Fig. 5E,F). Non-olaparib PARPIs, including niraparib, talazoparib, and rucaparib, provided rPFS benefit (HR: 0.70, 95% CI: 0.60–0.81, *P* < 0.001), but also have no OS benefit observed (HR: 0.98, 95% CI: 0.82–1.17, *P* = 0.800) (Fig. 5G,H). Moreover, the PSA-PFS was prolonged in patients with PARPIs (HR: 0.47, 95% CI: 0.36–0.61, *P* < 0.001) (Supplemental Digital Content Figure S9B, available at: http://links.lww.com/JS9/F126).

**Figure 6.**
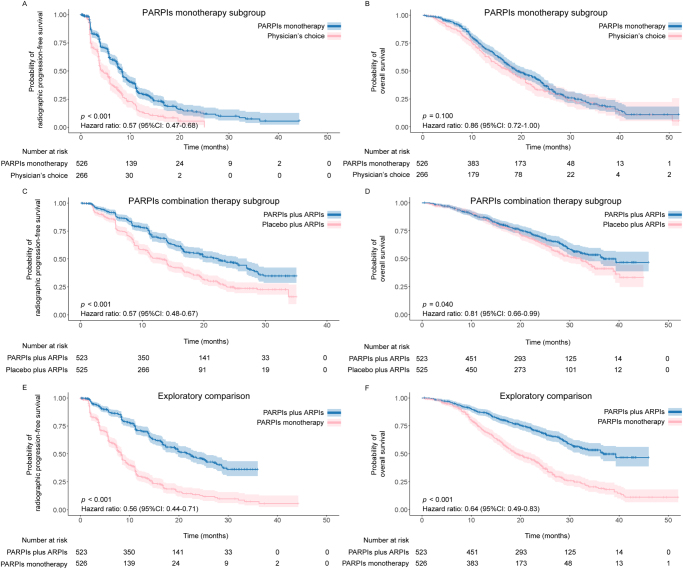
Kaplan-Meier curves of rPFS (A) and OS (B) in subgroup treated with PARPIs monotherapy versus Physician’s choice; Kaplan-Meier curves of rPFS (C) and OS (D) in subgroup treated with PARPIs plus ARPIs versus placebo plus ARPIs; Kaplan-Meier curves of rPFS (E) and OS (F) in subgroup treated with PARPIs plus ARPIs versus PARPIs monotherapy.

Abbreviations: ARPIs: Androgen receptor pathway inhibitors; CI: Confidence intervals; HR: Hazard ratio; OS: Overall survival; PARPIs: Poly(ADP-ribose) polymerase inhibitors; rPFS: Radiographic progression-free survival.

The survival benefits of PARPIs monotherapy (PARPIs *vs* Physician’s choice) or PARPIs combination therapy (PARPIs *vs* Placebo, with background ARPIs) were explored. Comparing with Physician’s choice treatment, including docetaxel chemotherapy and ARPIs, PARPIs monotherapy improved rPFS (HR: 0.57, 95% CI: 0.47–0.68, *P* < 0.001), but had no effect on OS (HR: 0.86, 95% CI: 0.72–1.00, *P* = 0.100) (Fig. 6A,B). Meanwhile, PARPIs combination therapy prolonged both rPFS (HR: 0.57, 95% CI: 0.48–0.67, *P* < 0.001), and OS (HR: 0.81, 95% CI: 0.66–0.99, *P* = 0.040) (Fig. 6C,D). Additionally, as an exploratory assessment, the effects of PARPIs mono- and combination therapy in the overall population were compared. Specifically, PARPIs combination therapy demonstrated better efficacy than monotherapy on rPFS (HR: 0.56, 95% CI: 0.44–0.71, *P* < 0.001), and OS (HR: 0.64, 95% CI: 0.49–0.83, *P* < 0.001) (Fig. 6E,F).

Please note: Reconstructed IPD for PARPIs monotherapy subgroup were extracted from PROfound and TRITON3; Reconstructed IPD for PARPIs combination therapy subgroup were extracted from PROpel, MAGNITUDE, and TALAPRO-2; OS data of MAGNITUDE, TALAPRO-2, and TRITON3 are immature.

### The safety of PARPIs in the overall population with HRR alteration

Any AEs, serious AEs, and grade ≥ 3 AEs were used to assess the safety of PARPIs in the overall population with HRR alteration. The incidences of any AEs between patients with PARPIs and control arms were similar (RR: 1.04, 95% CI: 1.02–1.06, *I^2^* = 12.3%, *P* = 0.3313) (Supplemental Digital Content Figure S10A, available at: http://links.lww.com/JS9/F126). The risk of serious AEs in PARPIs group was higher than control group (RR: 1.44, 95% CI: 1.23–1.68, *I^2^* = 0.0%, *P* = 0.4416) (Supplemental Digital Content Figure S10B, available at: http://links.lww.com/JS9/F126). Meanwhile, the incidence of grade ≥ 3 AEs was also higher in the PARPIs group (RR: 1.40, 95% CI: 1.20–1.63, *I^2^* = 60.1%, *P* = 0.0571) (Supplemental Digital Content Figure S10C, available at: http://links.lww.com/JS9/F126). Despite substantial heterogeneity detected, all directions and intervals of estimates for included trials were consistent in the sensitivity analyses (Supplemental Digital Content Figure S11A-C, available at: http://links.lww.com/JS9/F126).

### Publication bias

Contour-enhanced funnel plot were used to explore the potential publication bias for each analysis despite limited number of included studies. No significant publication bias was detected in the pooled rPFS and OS except for MAGNITUDE in the overall population with HRR alteration (Supplemental Digital Content Figure S12A,B, available at: http://links.lww.com/JS9/F126). Publication bias of PROpel was detected for PSA-PFS in the overall population (Supplemental Digital Content Figure S12C, available at: http://links.lww.com/JS9/F126). No significant publication bias was detected in subgroup analyses of rPFS and OS for clinical characteristics except MAGNITUDE (Supplemental Digital Content Figure S12D-I, available at: http://links.lww.com/JS9/F126). In subgroup analysis of HRR genes, several outliers were observed for rPFS and OS in *BRCA1/2* subgroup, suggesting potential publication bias (Supplemental Digital Content Figure S13A,D, available at: http://links.lww.com/JS9/F126). For *ATM* and *CHEK2* subgroups, no significant publication bias were detected (Supplemental Digital Content Figure S13B,C and E, available at: http://links.lww.com/JS9/F126). Additionally, pooled estimates of all safety outcomes did not indicate publication bias (Supplemental Digital Content Figure S13F-H, available at: http://links.lww.com/JS9/F126). The alignments between the pooled effect lines and the null effect regions in funnel plots were consistent with the findings of all forest plots.

## Discussion

Metastatic PCa remains one of the leading causes of cancer-related mortality in males worldwide, causing significant health burdens due to the clinical challenges in management^[2,29]^. The advanced and lethal stage of this condition, namely mCRPC, is characterized by rapid progression and poor prognosis despite the application of multidisciplinary treatment, including ADT, taxane chemotherapy, and novel targeted immune therapy^[30,31]^. Meanwhile, the resistance to existing ARPIs and chemotherapy emerges commonly, highlighting the necessity for more effective and personalized therapies within specific patient subsets^[32]^. In recent years, the advanced understanding of DNA repair pathway deficiencies in tumorigenesis has established several treatments in solid malignancies^[33–35]^. The clinical benefit of PARPIs has been demonstrated in ovarian and breast cancers, and emerging trials have reported various benefits of this targeted drug in mCRPC^[15,36,37]^.

PARPIs represent a promising option for mCRPC, especially for patients harboring HRR gene mutation, which render tumors particularly susceptible to synthetic lethality^[38,39]^. Recent phase III clinical trials have demonstrated survival benefits of PARPIs, including olaparib, niraparib, talazoparib, and rucaparib, in prolonging rPFS in mCRPC patients with HRR alterations^[4,5,15–21]^. However, each trial was limited by relatively small sample sizes of subgroup with specific HRR gene mutation. Meanwhile, diverse background therapies and mature outcomes of ongoing trials may bias the results^[5]^. These limitations have may obscure the overall therapeutic impact of PARPIs on patients with specific HRR gene mutation, highlighting the necessity of pooling data for robust clinical conclusions.

In this meta-analysis, we utilized subgroup data and reconstructed IPD from five pivotal phase III RCTs, and subsequently performed subgroup analyses of HRR gene and detailed survival analyses. Despite inherent biases in follow-up durations, background therapy, treatment regimens, and immature outcomes, our analysis yielded clinically meaningful results. The pooled estimates demonstrated improved rPFS in the overall population, highlighting the efficacy of PARPIs. Importantly, subgroup analyses of rPFS stratified by age, bone or visceral metastasis, previous taxane use, ECOG performance status, and geographic region showed consistent directions favoring the effects of PARPIs, suggesting wide-ranging applicable potential across diverse clinical subgroups. These findings align with previous studies but expand upon them by systematically integrating subgroup and reconstructed IPD analyses, which may enhance their generalizability and applicability in clinical setting^[40,41]^.

We found PARPIs have no effect on rPFS and OS in *ATM*-altered patients, and did not prolong the OS in *CDK12* subgroup, highlighting that these HRR-altered tumors may not suffer synthetic lethality when PARP inhibition used alone, possibly due to residual end-joining repair capacity or functional redundancy. In clinical setting, these results may suggest that administrating PARPIs in *ATM*-mutated mCRPC should be avoided. Meanwhile, different outcomes between non-*BRCA* genes may emphasize the heterogeneity of HRR alteration and the importance of prospective biomarker validation. On the other hand, pooled estimates demonstrated that PARPIs significantly reduced the risk of death in the overall population and several subgroups, include *BRCA1/2* and *CHEK2*, unlike the OS outcomes reported in previous studies^[15,21]^. This discrepancy may be attributed to the small sample size and event counts of HRR mutation patients. Thus, we further explore the survival benefit of PARPIs using reconstructed IPD.

Similar to the results of forest plots, significant rPFS benefits of PARPIs in both overall population or *BRCA1/2* subgroup were observed in survival analyses, confirming the favorable efficacy of PARPIs. Meanwhile, a novel finding from reconstructed IPD was the long-term OS benefit of PARPIs in patients with *BRCA1/2* mutations. This result is different with previous individual trial that reported improved prognosis except for OS of *BRCA1/2* subgroup, and the discrepancy maybe attributed to limited sample sizes of *BRCA1/2* patients^[21]^. We further performed individual analyses of olaparib and other PARPIs in the overall population, and found that both olaparib and the other PARPIs improved the rPFS, which were consistent with the pooled estimates in our forest plot. Moreover, the PARPIs also prolonged PSA-PFS in the overall population and *BRCA1/2* subgroup.

By aggregating and reconstructing individual-level survival data across multiple studies, we substantially increased the sample sizes of the overall population and specific HRR gene subgroup. This method enhances the detection of survival differences that is lacked in individual studies alone^[26,28]^. This method allows us to explore potential clinical issues with more flexibility when original IPD is not easy to obtain. For example, we explore the survival benefits of PARPIs monotherapy (PARPIs *vs* Physician’s choice) or PARPIs combination therapy (PARPIs *vs* Placebo, with background ARPIs). Comparing with PARPIs monotherapy, PARPIs combination therapy increased both rPFS and OS, suggesting that PARPIs with background ARPIs might be the better option for mCRPC.

We also observed moderate-to-substantial heterogeneity in certain pooled outcomes, particularly for rPFS. Further investigation suggested that the central review data from the MAGNITUDE trial may contributed significantly to the heterogeneity. Interestingly, when rPFS data by investigator review were used instead, the heterogeneity decreased remarkably. This tendency may reflect the differences of radiographic assessment in post-therapy phase^[42,43]^. Moreover, the PROpel trial showed substantial heterogeneity in some subgroup analyses, particularly in the *BRCA1/2* subgroup for OS and PSA-PFS. This might be attributed to the small sample size of *BRCA1/2* subgroup in PROpel which may lead to unstable estimates and outlier. Therefore, we presented the analyses excluding heterogeneous study to evaluate the robustness of these findings.

This study has several limitations that warrant consideration. First, inherent limitations of meta-analyses include potential biases arising from heterogeneity among included studies, variations in treatment protocols, differences in patient populations, and inconsistent reporting of outcomes. Second, IPD inherently involves approximations derived from published Kaplan–Meier curves rather than directly obtained original data, and some results come from the interim analysis or immature outcomes, which may introduce certain inaccuracies and weakened the power of several results. Particularly, the OS data from MAGNITUDE, TALAPRO-2, and TRITON3 remain immature, with relatively few death events and potential right censoring in interim KM curves, causing discrepancy to pooled result. Third, our analysis included only five phase III RCTs. Thus, findings related to less frequent HRR gene mutations, such as *ATM, CDK12*, and *CHEK2*, may be less robust, due to smaller subgroup sizes and events. Additionally, although meta-regression suggested that PARPIs type and background therapy have no statistically significant influence on rPFS and OS, different PARPIs protocol may introduce potential heterogeneity. Lastly, differences from crossover design in trials may influence the OS outcomes observed, and these were not fully accounted for in our analysis.

## Conclusions

PARPIs treatment demonstrates significant clinical efficacy and favorable safety profile in mCRPC patients with HRR alteration. PARPIs improve the survival in patients with *BRCA1/2* mutation but have no effect in patients with *ATM* mutation. Comparing with monotherapy, the combination of PARPIs with ARPIs provides greater survival benefit in the overall population. Future investigation should validate these findings in real-world settings.

## Supplementary Material

## Data Availability

The datasets analyzed during the current study are available from the corresponding author on reasonable request.

## Research registration unique identifying number (UIN)

PROSPERO (CRD420251042411).
