# Supplementary material for: Exploring the efficacy of PARP inhibitors in metastatic castration-resistant prostate cancer with homologous recombination repair alteration: a meta-analysis based on subgroups and reconstructed individual patient data
Source: Int J Surg. 2025 Sep 19;112(1):1787–99. doi: 10.1097/JS9.0000000000003338 (PMC12825753; doi:10.1097/JS9.0000000000003338)

**Figure S1.** Risk of bias assessment using RoB2 tool.

Abbreviations: RoB: Risk of bias.


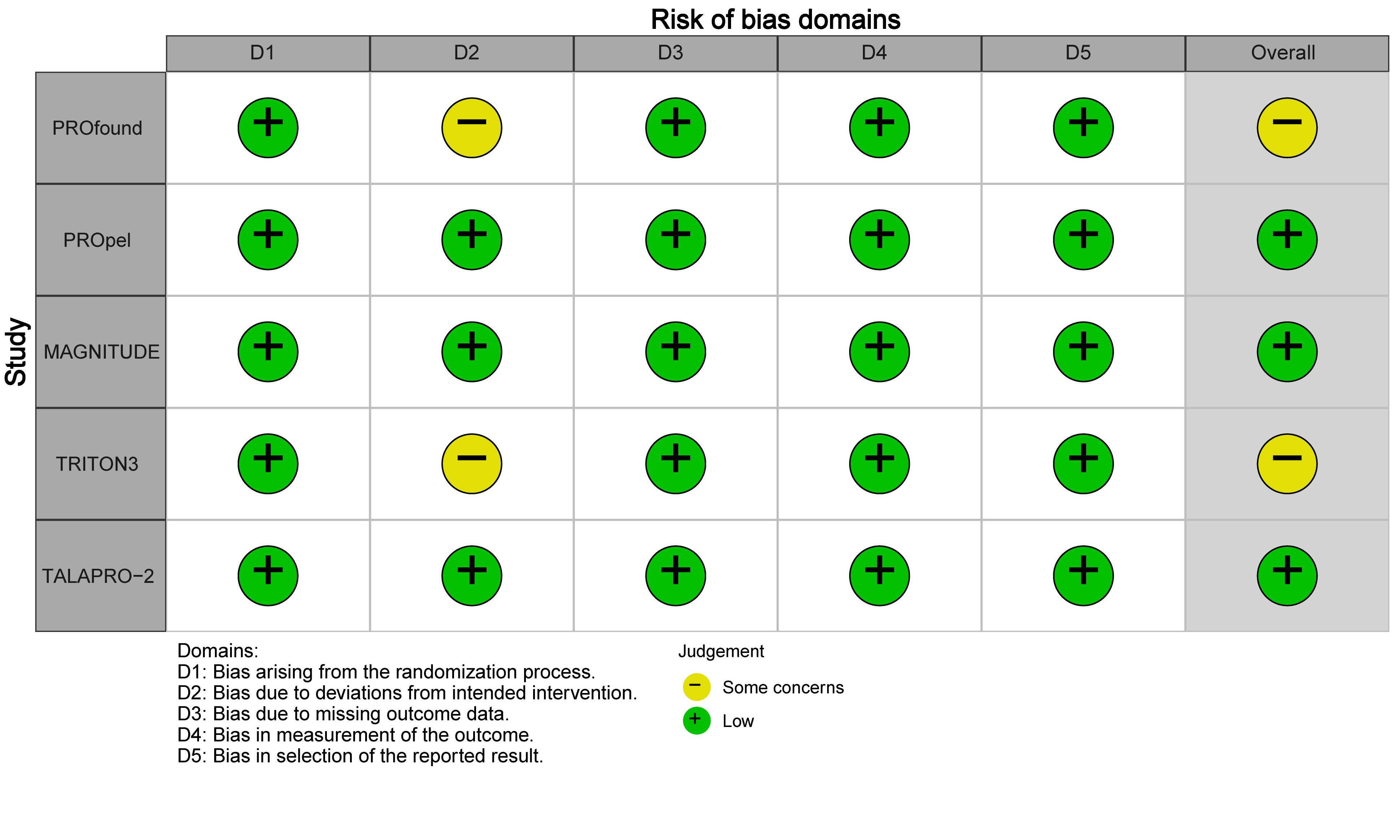


**Figure S2.** Leave‑one‑out sensitivity analyses of rPFS in HRR-altered overall population (A), when excluding heterogeneous study (B), and when including the investigator-reviewed rPFS results from heterogeneous study (C); The sensitivity analyses of OS in HRR-altered overall population (D).

Abbreviations: CI: Confidence intervals; HR: Hazard ratio; OS: Overall survival; rPFS: Radiographic progression-free survival.


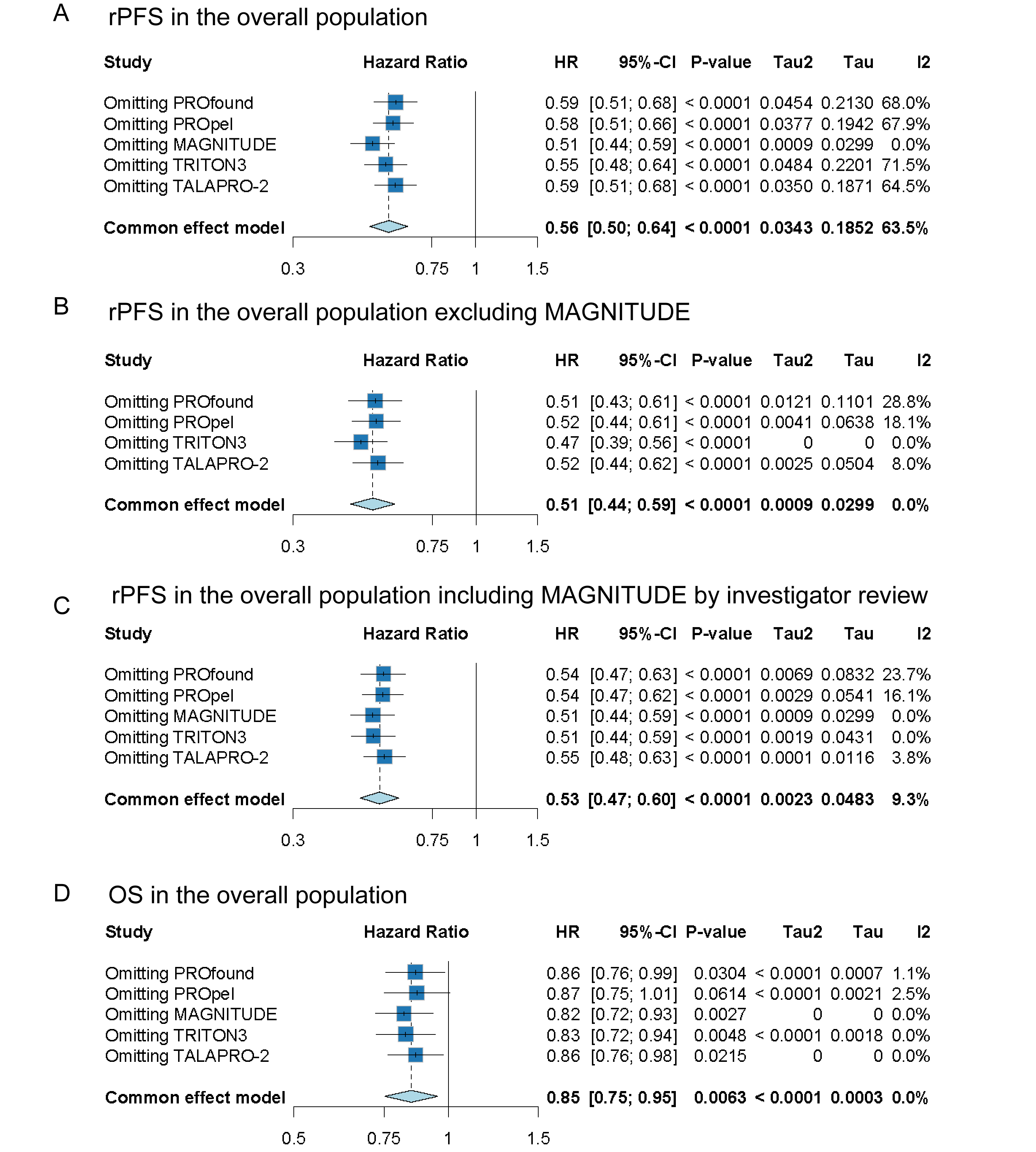


**Figure S3.** Forest plots showing the effects of PARPIs on rPFS in HRR-altered overall population when excluding heterogeneous study (A), and when including the investigator-reviewed rPFS results from heterogeneous study (B); The effects of PARPIs on PSA-PFS in HRR-altered overall population.

Abbreviations: CI: Confidence intervals; HR: Hazard ratio; OS: Overall survival; PARPIs: Poly(ADP-ribose) polymerase inhibitors; rPFS: Radiographic progression-free survival; PSA-PFS: Prostate-specific antigen progression-free survival.


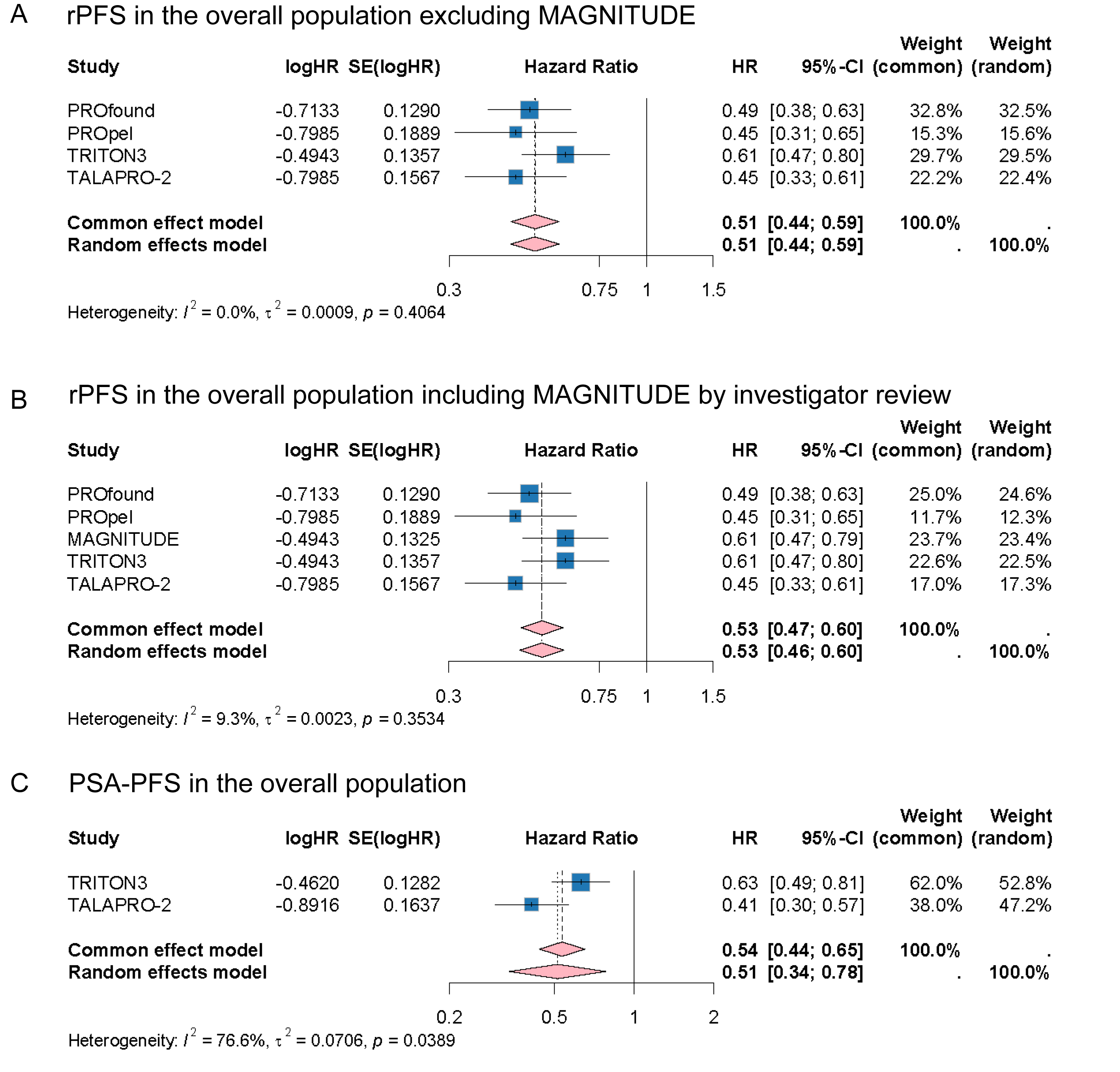


**Figure S4.** Leave‑one‑out sensitivity analyses of rPFS in subgroups at age < 65 (A), age ≥ 65 (B), with only bone metastasis (C) and with only visceral metastasis (D).

Abbreviations: CI: Confidence intervals; HR: Hazard ratio; rPFS: Radiographic progression-free survival.


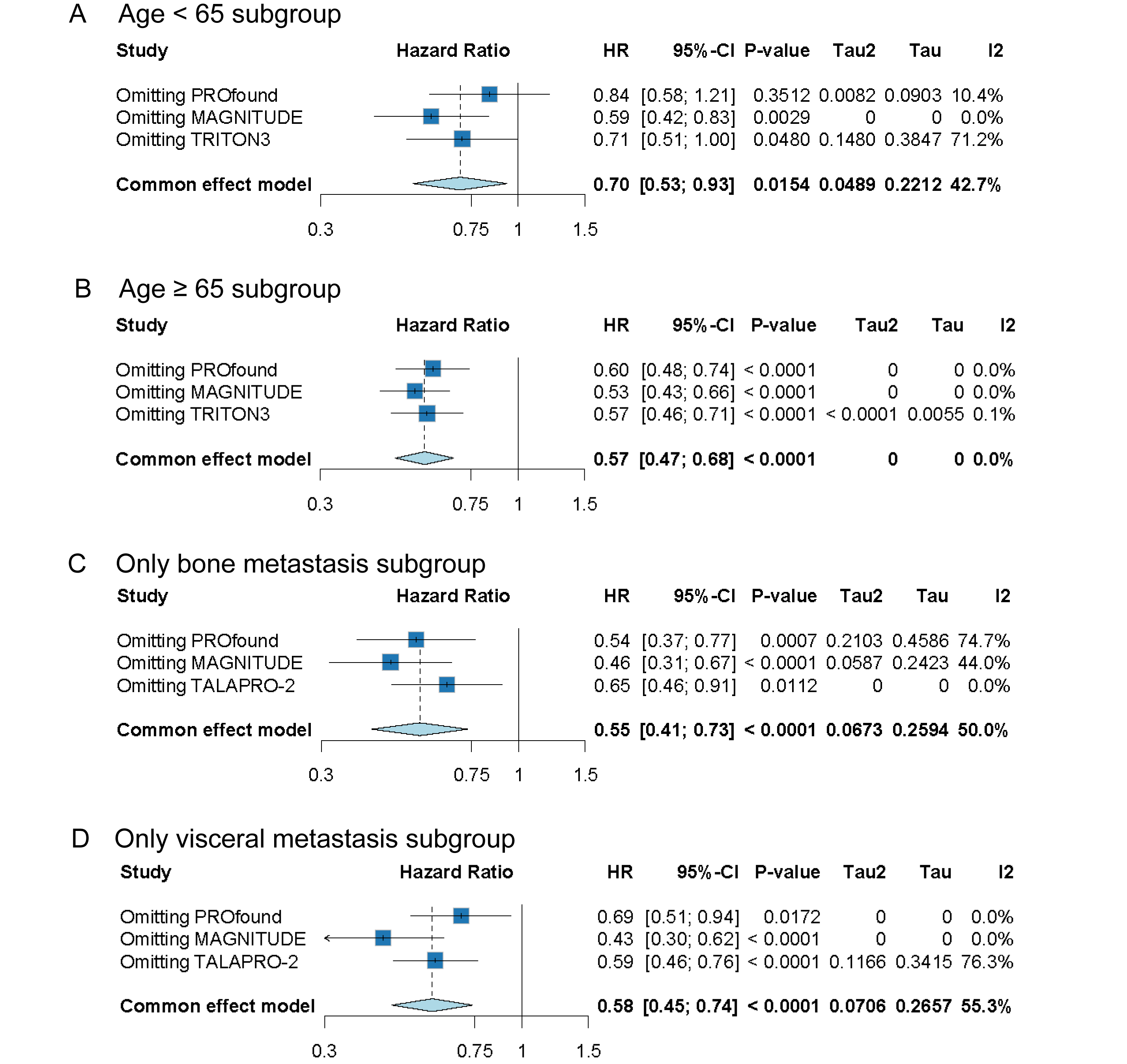


**Figure S5.** Forest plots showing the effects of PARPIs on rPFS in subgroups defined by ECOG score (A); Leave‑one‑out sensitivity analyses of rPFS in subgroups with ECOG score 0 (B) and ECOG score 1 (C).

Abbreviations: CI: Confidence intervals; ECOG: Eastern Cooperative Oncology Group; HR: Hazard ratio; PARPIs: Poly(ADP-ribose) polymerase inhibitors; rPFS: Radiographic progression-free survival.


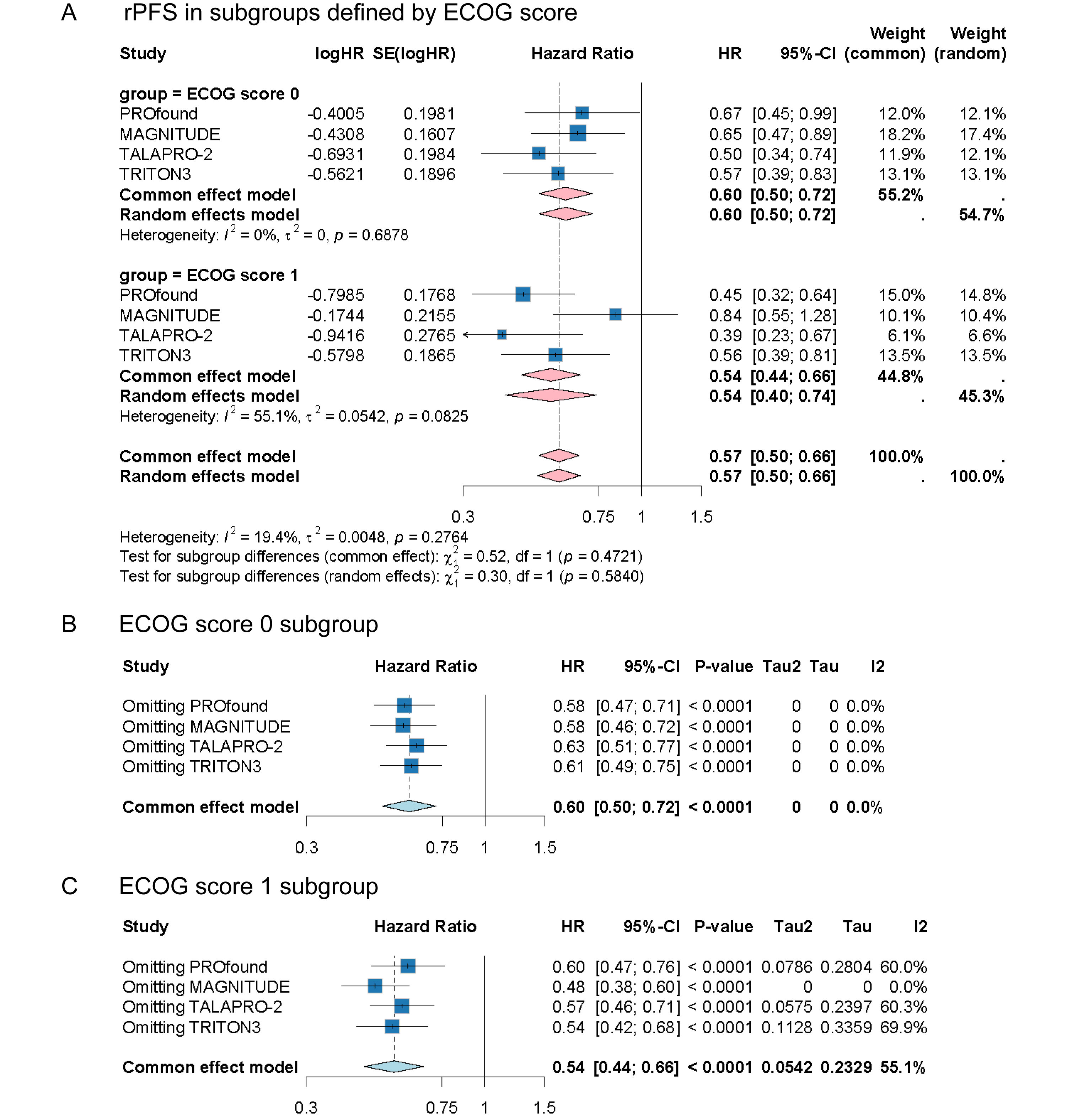


**Figure S6.** Forest plots showing the effects of PARPIs on rPFS in subgroups defined by ECOG score when excluding heterogeneous study (A), and in subgroups defined by geographic region of patients (B).

Abbreviations: CI: Confidence intervals; Eastern Cooperative Oncology Group; HR: Hazard ratio; PARPIs: Poly(ADP-ribose) polymerase inhibitors; rPFS: Radiographic progression-free survival.


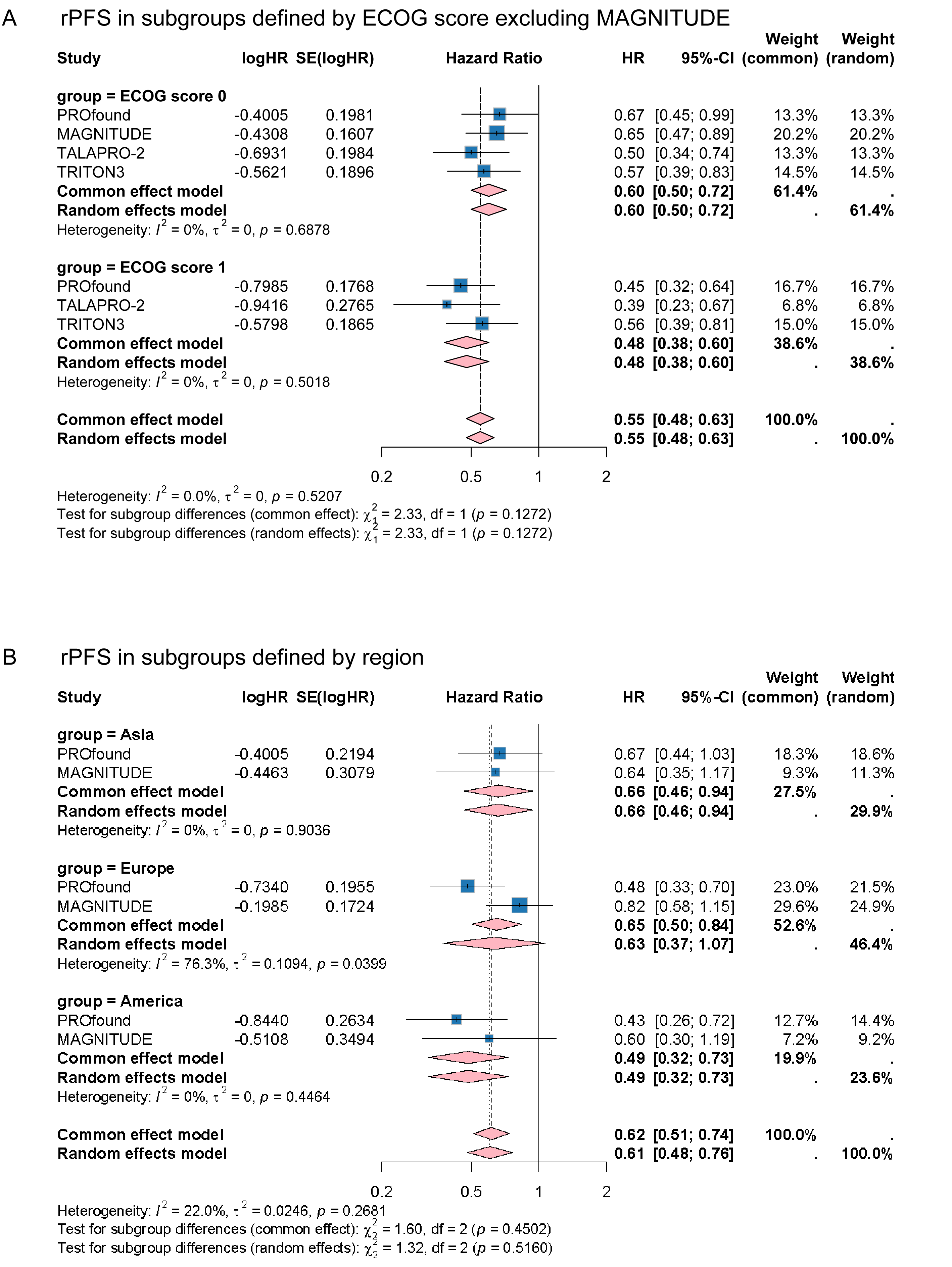


**Figure S7.** Forest plots showing the effects of PARPIs on rPFS in subgroup with BRCA1/2 mutation stratified by PARPIs type (Olaparib *vs* Non-olaparib) (A); The effects of PARPIs on PSA-PFS in subgroup with BRCA1/2 mutation (B), and when excluding heterogeneous study(C); The effects of PARPIs on OS in subgroup with BRCA1/2 mutation (D);

Abbreviations: CI: Confidence intervals; HR: Hazard ratio; OS: Overall survival; PARPIs: Poly(ADP-ribose) polymerase inhibitors; PSA-PFS: Prostate-specific antigen progression-free survival; rPFS: Radiographic progression-free survival.


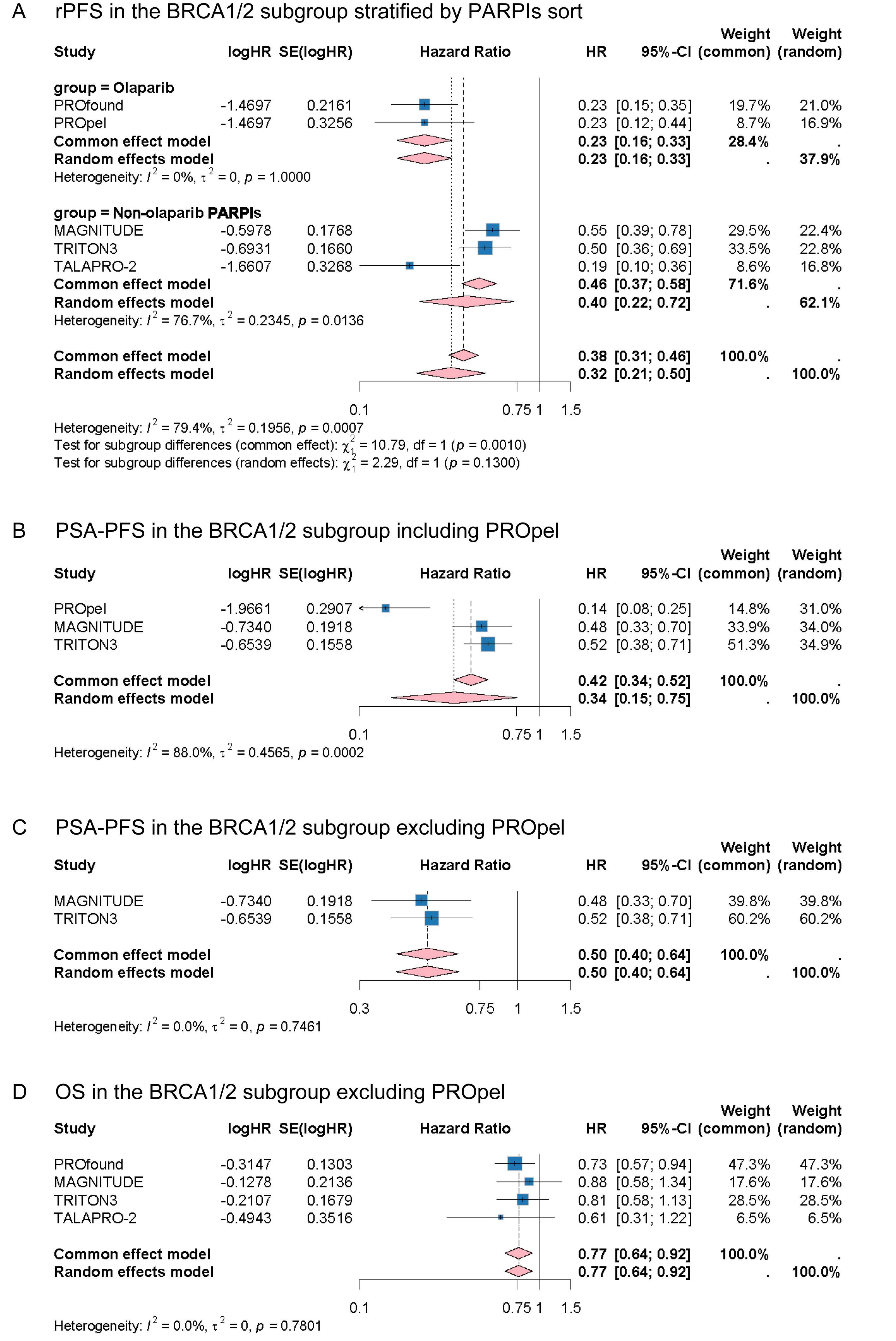


**Figure S8.** Leave‑one‑out sensitivity analyses of rPFS in subgroups with *BRCA1/2* mutation (A), *ATM* mutation (B), *CDK12* mutation and *CHEK2* mutation (D); The sensitivity analyses of PSA-PFS in subgroups with *BRCA1/2* mutation (E); The sensitivity analyses of OS in subgroups with *BRCA1/2* mutation (F) and *ATM* mutation (G).

Abbreviations: ATM: Ataxia Telangiectasia Mutated gene; BRCA1/2: Breast Cancer 1/2 gene; CDK12: Cyclin-Dependent Kinase 12 gene; CHEK2: Checkpoint Kinase 2 gene; CI: Confidence intervals; HR: Hazard ratio; HRR: Homologous recombination repair; OS: Overall survival; PSA-PFS: Prostate-specific antigen progression-free survival; rPFS: Radiographic progression-free survival.


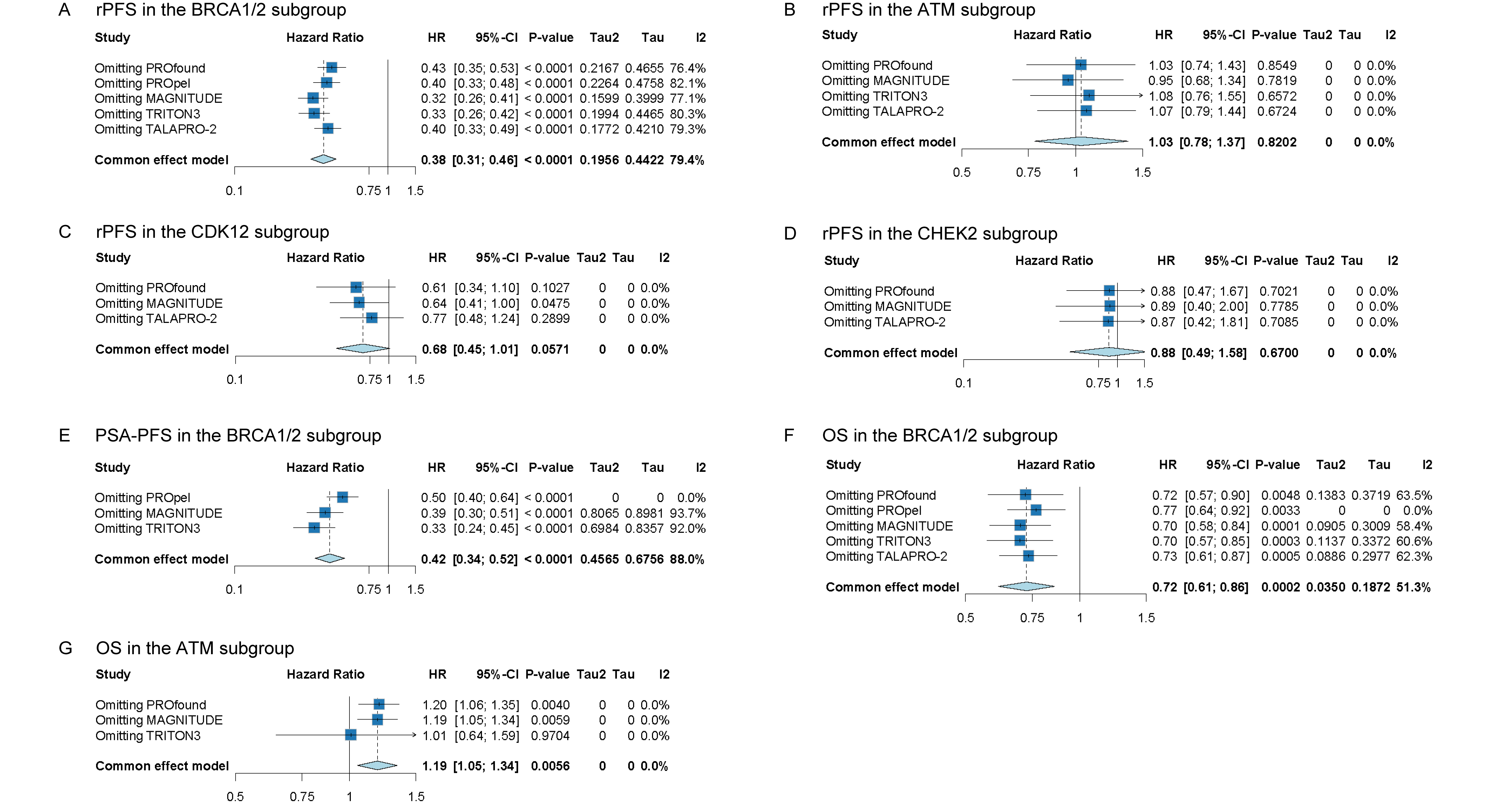


**Figure S9.** Kaplan-Meier curve of PSA-PFS in HRR-altered overall population (A), and in *BRCA1/2* mutation subgroup (B) treated with PARPIs versus control.

Abbreviations: BRCA1/2: Breast Cancer 1/2 gene; CI: Confidence intervals; HR: Hazard ratio; HRR: Homologous recombination repair; PARPIs: Poly(ADP-ribose) polymerase inhibitors; PSA-PFS: Prostate-specific antigen progression-free survival.

Please note: Reconstructed IPD were extracted from PROpel and TRITON3.


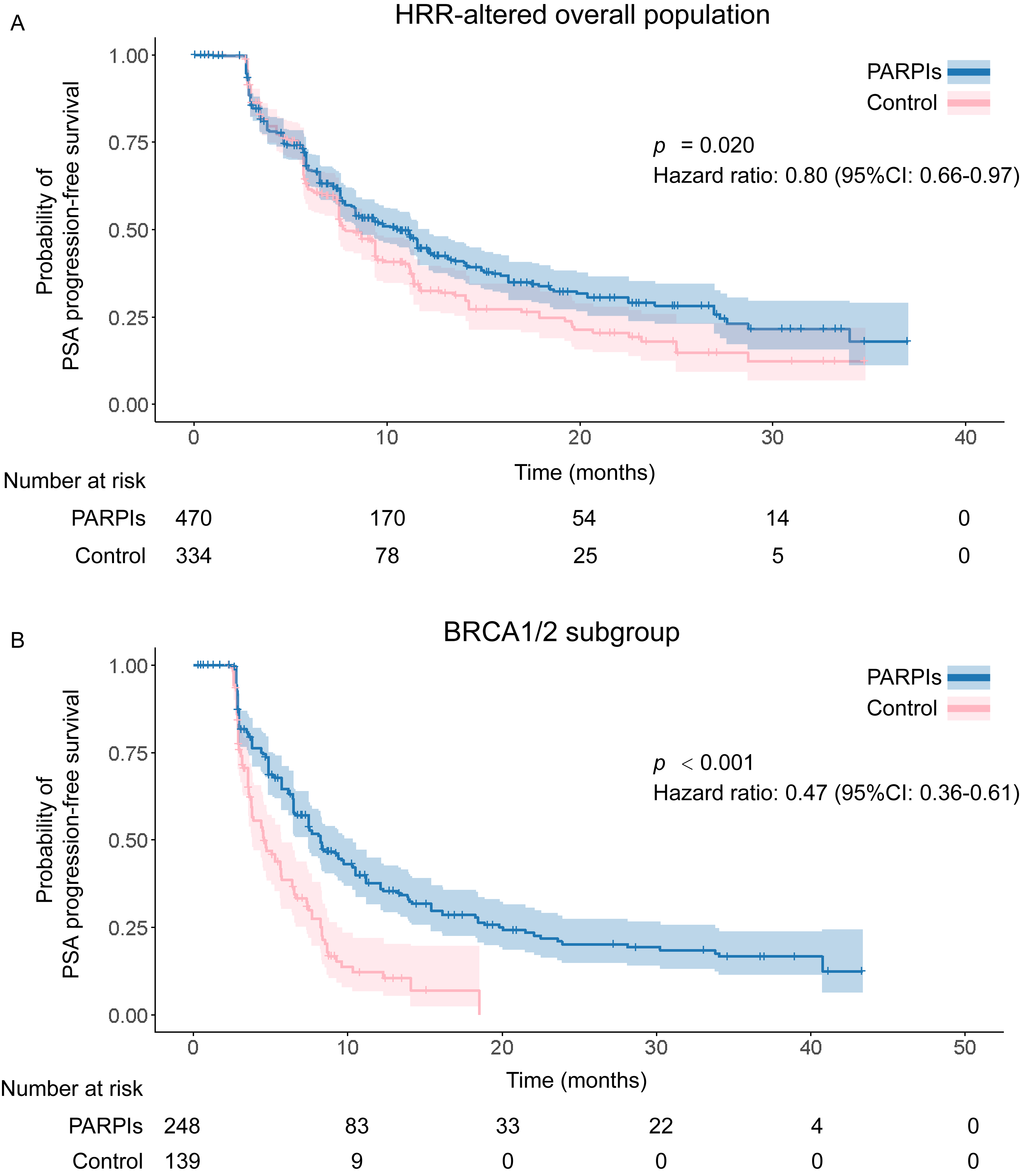


**Figure S10.** Forest plots profiling the safety of PARPIs in HRR-altered overall population by any AEs (A), serious AEs (B) and grade ≥ 3 AEs (C).

Abbreviations: AEs: Adverse events; CI: Confidence intervals; HR: Hazard ratio; PARPIs: Poly(ADP-ribose) polymerase inhibitors;


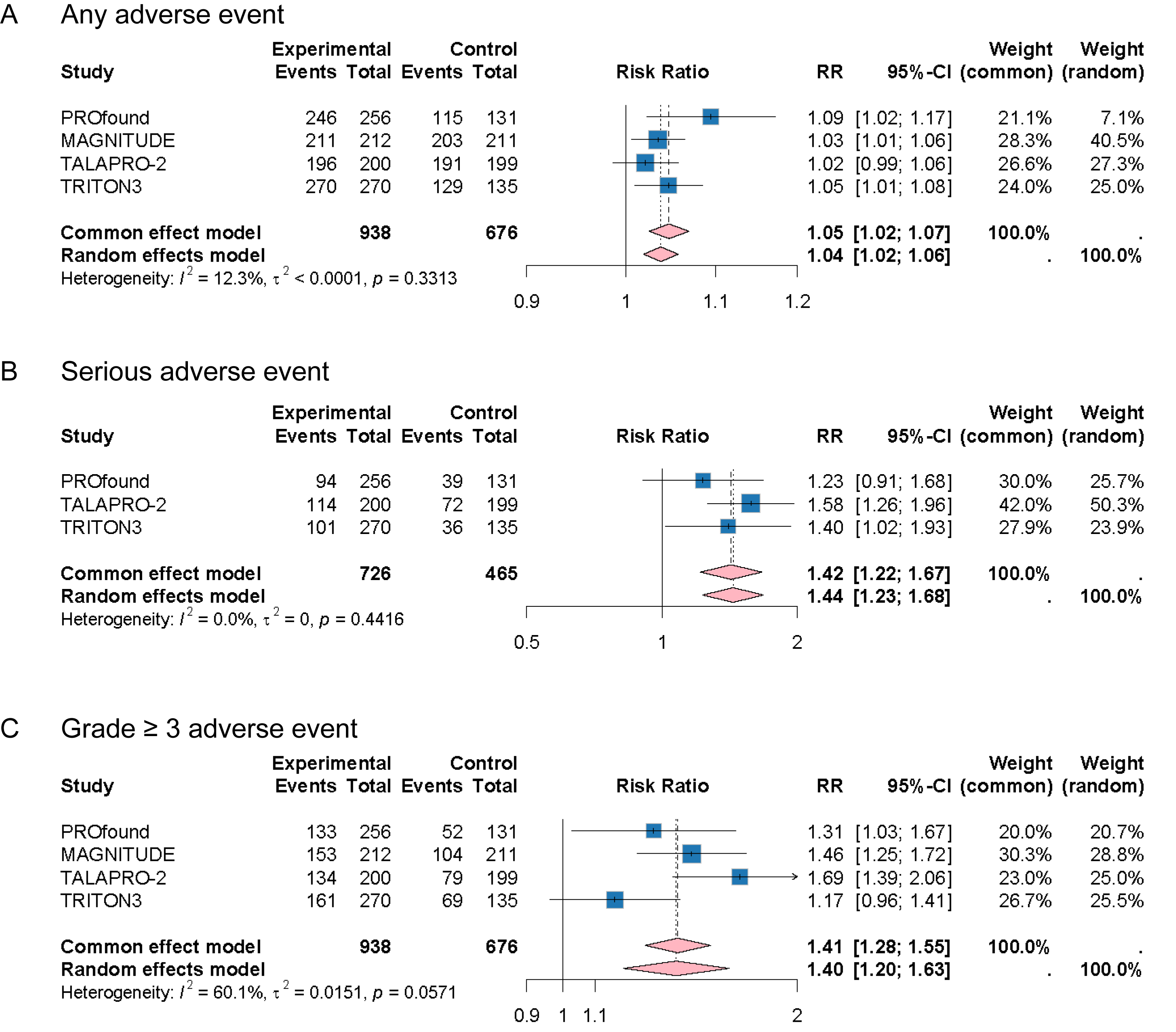


**Figure S11.** Leave‑one‑out sensitivity analyses of AEs (A), serious AEs (B) and grade ≥ 3 AEs.

Abbreviations: AEs: Adverse events; CI: Confidence intervals; HR: Hazard ratio; PARPIs: Poly(ADP-ribose) polymerase inhibitors;


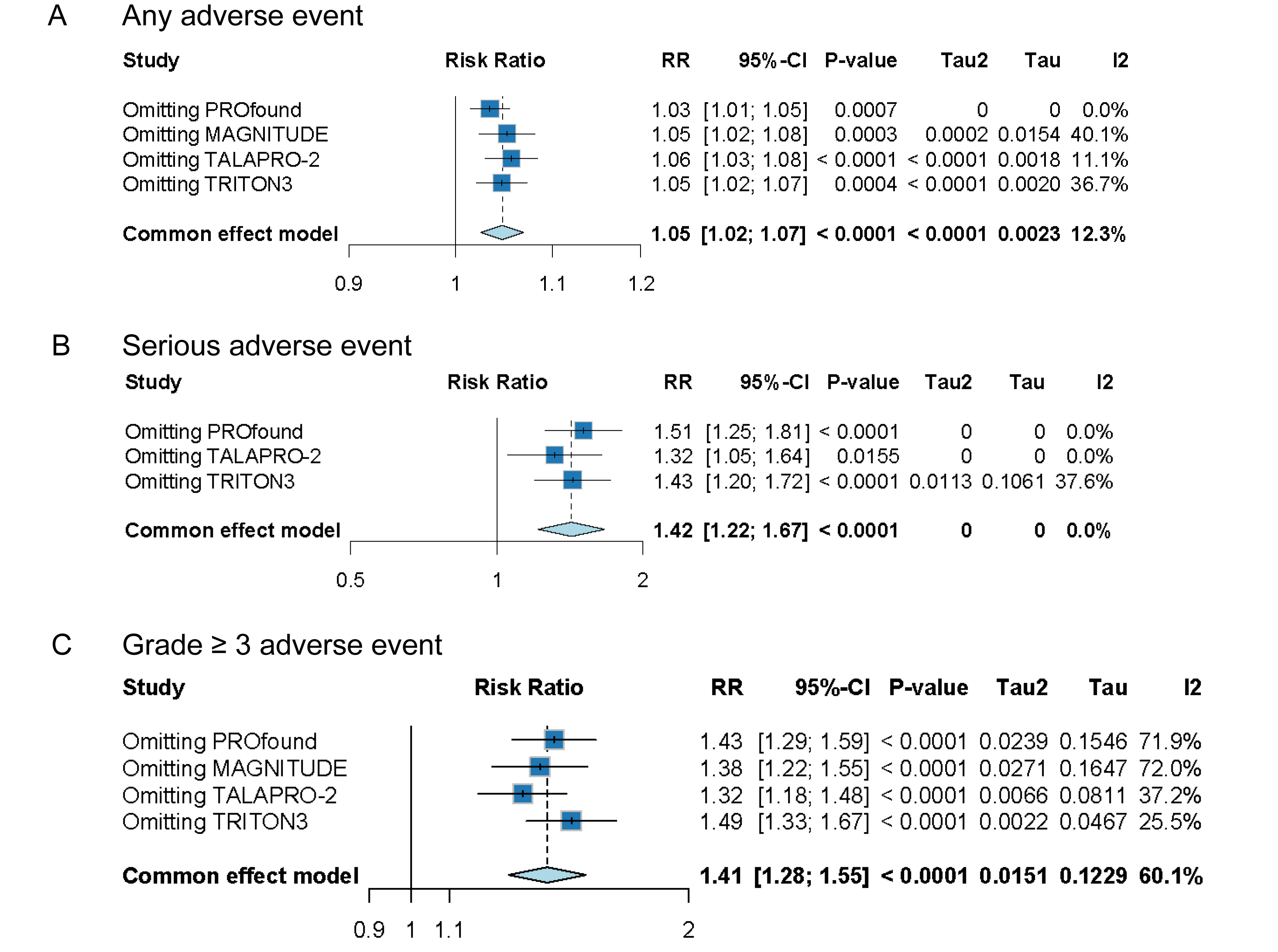


**Figure S12.** Contour‑enhanced funnel plots for rPFS and OS in HRR-altered overall population (A to C) and clinical subgroups (D to I).

Abbreviations: Eastern Cooperative Oncology Group; HR: Hazard ratio; HRR: Homologous recombination repair; OS: Overall survival; PSA-PFS: Prostate-specific antigen progression-free survival; rPFS: Radiographic progression-free survival.


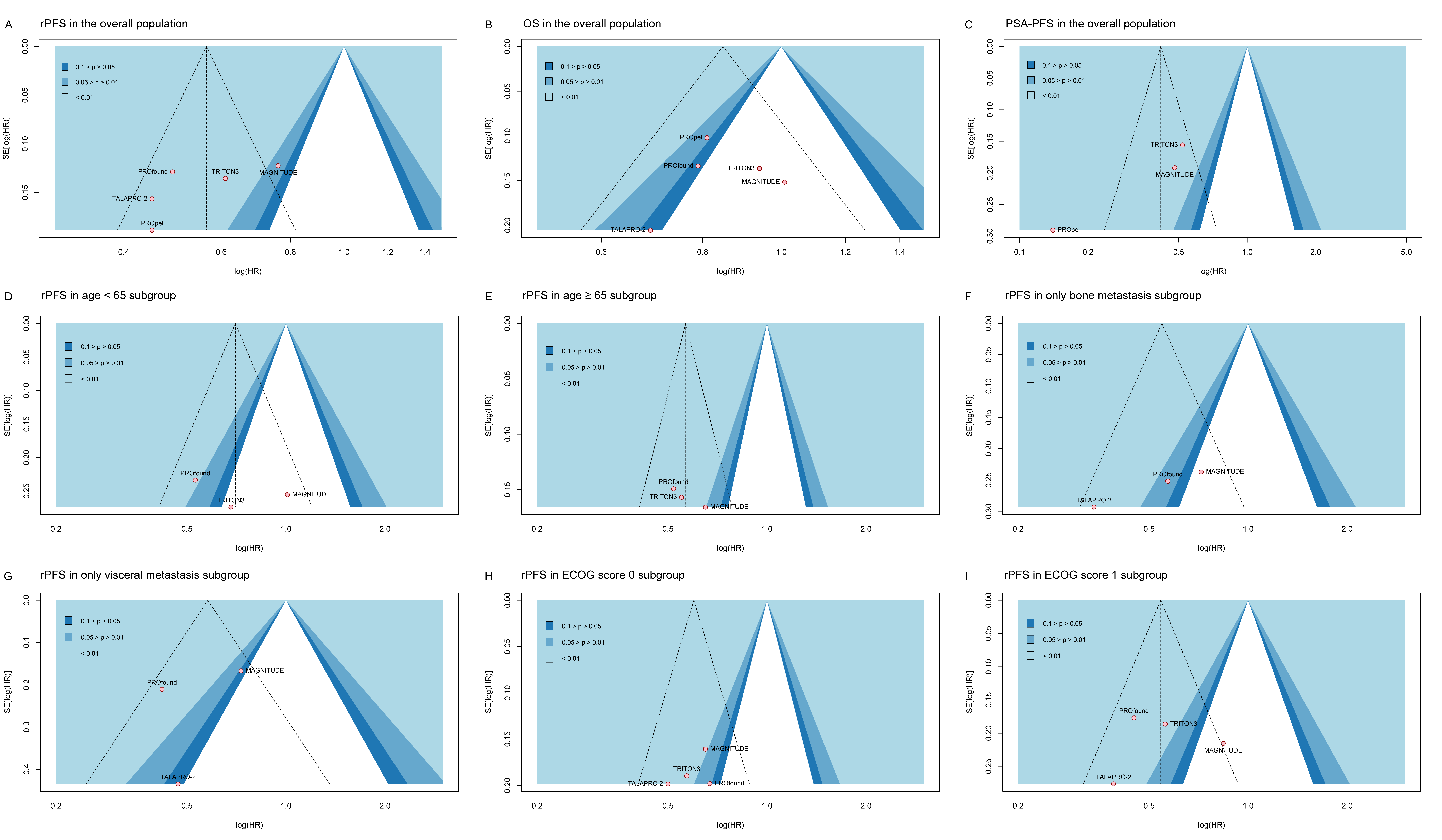


**Figure S13.** Contour‑enhanced funnel plots for rPFS and OS in HRR-altered overall in subgroups with specific HRR gene mutation (A to E); Contour‑enhanced funnel plots for AEs (F), serious AEs (G) and grade ≥ 3 AEs (H).

Abbreviations: AEs: Adverse events; ATM: Ataxia Telangiectasia Mutated gene; BRCA1/2: Breast Cancer 1/2 gene; CDK12: Cyclin-Dependent Kinase 12 gene; CHEK2: Checkpoint Kinase 2 gene; HR: Hazard ratio; HRR: Homologous recombination repair; OS: Overall survival; rPFS: Radiographic progression-free survival.


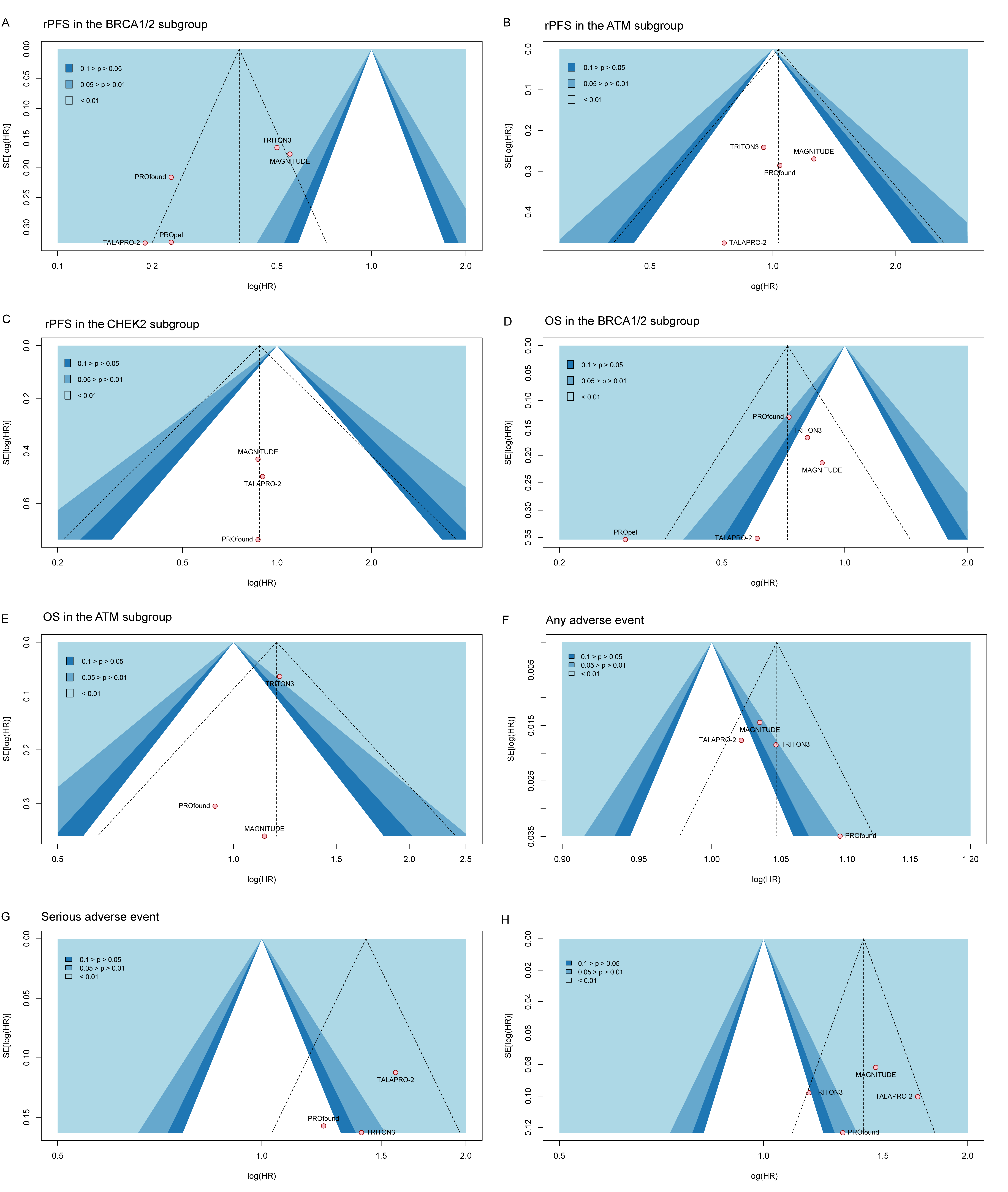

Supplement: Supplementary file 1 [file js9-112-1787-001.docx]
