# Supplementary material for: Exploring the efficacy of PARP inhibitors in metastatic castration-resistant prostate cancer with homologous recombination repair alteration: a meta-analysis based on subgroups and reconstructed individual patient data
Source: Int J Surg. 2025 Sep 19;112(1):1787–99. doi: 10.1097/JS9.0000000000003338 (PMC12825753; doi:10.1097/JS9.0000000000003338)
Supplement: Supplementary file 2 [file js9-112-1787-002.doc]

Table S1. Results of meta-regression.

| Varibles | rPFS | | | | | |  | OS | | | | | |
| --- | --- | --- | --- | --- | --- | --- | --- | --- | --- | --- | --- | --- | --- |
|  | Estimate (β) | τ² | *I²* (%) | R² (%) | QM | *p* (QM) |  | Estimate (β) | τ² | *I²* (%) | R² (%) | QM | *p* (QM) |
| Age | -0.063 | 0.050 | 70.40 | 0.00 | 0.068 | 0.794 |  | 0.012 | 0.000 | 0.00 | 0.00 | 0.008 | 0.927 |
| PARPIs type | 0.244 | 0.028 | 57.54 | 17.63 | 1.408 | 0.236 |  | 0.123 | 0.000 | 0.02 | 0.00 | 1.021 | 0.312 |
| Background therapy | -0.005 | 0.052 | 71.00 | 0.00 | 0.001 | 0.983 |  | 0.025 | 0.000 | 0.010 | 0.00 | 0.039 | 0.843 |

Abbreviation: ARPIs: Androgen receptor pathway inhibitors; OS: Overall survival; PARPIs: Poly(ADP-ribose) polymerase inhibitors; rPFS: Radiographic progression-free survival.

Please note: PARPIs type included Olaparib and non-olaparib PARPIs (Reference: Olaparib); Background therapy included PARPIs monotherapy without background therapy and PARPIs plus background ARPIs (Reference: PARPIs monotherapy).
